# Effect of Physiotherapeutic Interventions on Biomarkers of Neuropathic Pain: A Systematic Review of Preclinical Literature

**DOI:** 10.1016/j.jpain.2022.06.007

**Published:** 2022-06-26

**Authors:** Luis Matesanz-García, Annina B. Schmid, Julio Eduardo Cáceres-Pajuelo, Ferran Cuenca-Martínez, Alberto Arribas-Romano, Yeray González-Zamorano, Carlos Goicoechea-García, Josué Fernández-Carnero

**Affiliations:** * Escuela Internacional de Doctorado, Department of Physical Therapy, Occupational Therapy, Rehabilitation and Physical Medicine, Universidad Rey Juan Carlos, Alcorcón, Spain; † Departamento de Fisioterapia, Centro Superior de Estudios Universitarios La Salle, Universidad Autónoma de Madrid, Madrid, Spain; ‡ Nuffield Department for Clinical Neurosciences, University of Oxford, Oxford, United Kingdom.; § Kapalua Fisioterapia S.L., Madrid, Spain; ¶ Exercise Intervention for Health Research Group (EXINH-RG), Department of Physiotherapy, University of Valencia, Valencia, Spain; ǁ Department of Physical Therapy, Occupational Therapy, Rehabilitation and Physical Medicine, Rey Juan Carlos University, Madrid, Spain; # Grupo de Investigaticón de Neurorrehabilitación del Daño Cerebral y los Trastornos del Movimiento (GINDAT), Facultad de Ciencias Experimentales, Universidad Francisco de Vitoria, Pozuelo de Alarcón, Madrid, Spain; ** Departament Basic Health Sciences Rey Juan Carlos University, Madrid, Spain; †† Motion in Brains Research Group, Institute of Neuroscience and Sciences of the Movement (INCIMOV), Centro Superior de Estudios Universitarios La Salle, Universidad Autónoma de Madrid, Madrid, Spain; ‡‡ Grupo Multidisciplinar de Investigaticón y Tratamiento del Dolor, Grupo de Excelencia Investigadora URJC-Banco de Santander, Madrid, Spain; §§ La Paz Hospital Institute for Health Research, IdiPAZ, Madrid, Spain

**Keywords:** Neuropathic pain, physical therapy modalities, animal model, preclinical study, biological factor, pain measurement

## Abstract

The purpose of this systematic review was to evaluate the effects of physiotherapeutic interventions on biomarkers of neuropathic pain in preclinical models of peripheral neuropathic pain (PNP). The search was performed in Pubmed, Web of Science, EMBASE, Cochrane, Cinhal, Psycinfo, Scopus, Medline, and Science Direct. Studies evaluating any type of physiotherapy intervention for PNP (systemic or traumatic) were included. Eighty-one articles were included in this review. The most common PNP model was chronic constriction injury, and the most frequently studied biomarkers were related to neuro-immune processes. Exercise therapy and Electro-acupuncture were the 2 most frequently studied physiotherapy interventions while acupuncture and joint mobilization were less frequently examined. Most physiotherapeutic interventions modulated the expression of biomarkers related to neuropathic pain. Whereas the results seem promising; they have to be considered with caution due to the high risk of bias of included studies and high heterogeneity of the type and anatomical localization of biomarkers reported. The review protocol is registered on PROSPERO (CRD42019142878).

## Introduction

Neuropathic pain (NP) is defined as pain caused by a lesion or a disease of the somatosensory system^1^ and is estimated to affect between 6.9 and 10% of the general population.^2,3^ Peripheral neuropathic pain is becoming more prevalent due to an aging world population, the rising impact of diabetes mellitus as well as higher survival rates of cancer and the implications of chemotherapy.^4^ Management of NP remains challenging, as many patients do not experience adequate pain relief.^5-8^

Treatment of neuropathic pain usually focuses on symptom management.^9^ Nonsurgical interventions are recommended as first-line treatments for patients with neuropathic pain.^10^ Among the nonsurgical interventions, the Neuropathic Pain Special Interest Group of the International Association for the Study of Pain recommends pharmacology as first-line treatment.^5,11^ However, efficacy is limited ^12^ with often unacceptable side effects.^12-14^

Over the past decade, the role of Physiotherapy and physical activity has gained increasing interest in the treatment of neuropathic pain.^15^ Several studies have been published on the efficacy of physiotherapy on peripheral neuropathic pain resulting from systemic^16^ or focal nerve damage.^17,15^ In addition several guidelines propose active exercise as a treatment option for neuropathic pain.^18,19^ Although some studies suggest that physiotherapy provides significant improvements in pain, quality of life and disability in patients with peripheral neuropathies and neuropathic pain,^20,21^ other studies did not report similar findings^15^ and the mixed quality of studies prevents firm conclusions.^15^ Whereas human studies evaluating physiotherapy for neuropathic pain focus on improving pain, function and quality of life, the mechanisms by which physiotherapy interventions work remains poorly understood. A better understanding of the mechanisms of action of physiotherapy would help the selection of the most promising disease modulating physiotherapy interventions for future clinical trials.

The body of literature exploring the mechanisms of action of physiotherapeutic interventions using preclinical models has grown substantially over the past years. The main objective of this systematic review is therefore to summarize this literature by assessing the effect of physiotherapeutic interventions on biomarkers of neuropathic pain in pre-clinical models.

## Methods

This systematic review was conducted following the guidelines of the Systematic Review Center for Laboratory Animal Experimentation (SYRCLE), the Cochrane Handbook for Systematic Review of Intervention,^22^ the original guide “Preferred Reporting Items for Systematic Reviews, PRISMA” and the most recent update from 2021.^23^ The protocol has been prospectively registered in the International Prospective Register of Systematic Reviews (PROSPERO, CRD42019142878).

### Literature Search

A systematic search was developed following the step-by-step guide suggested by Leenaars et al.^24^ The following databases were searched from inception to 13th January 2020 and updated in February 2022: MEDLINE EMBASE, CINAHL, SCOPUS, Web of Science, PubMed, Cochrane library and PsycINFO. The search strategy is described in Appendix 1.

### Selection Criteria

#### Types of Studies

Original animal studies reporting the effect of physiotherapeutic interventions compared to a control group on peripheral neuropathic pain were included. Case studies, cross-over studies, and studies without a separate control group were excluded. Letters, reports, or abstracts from congresses were not included. Only articles with access to the full-text in English and Spanish language were included.

#### Animal Models

In-vivo animal models of neuropathic pain induced by both systemic (eg, diabetic or chemotherapy induced neuropathy) and focal nerve injury (eg, nerve ligation, crushing or transection) were included. We excluded studies where due to the model or validation tests (eg, sensory thresholds), we could not ascertain that the animals had developed neuropathic pain. We also excluded studies with animals with co-morbidities (eg, pre-ische-mic physiologic conditions such as ischemic injury) and studies that evaluated the prevention rather than the treatment of already existing neuropathic pain.

#### Interventions

We included any physiotherapy intervention (eg, exercise, acupuncture, electro-acupuncture, joint mobilization, neural mobilization, physical agents), independent of timings and dosage. Studies evaluating invasive treatments (eg, radiofrequency or spinal stimulation) or pharmacological treatments were excluded.

#### Comparator

The control population was defined as a cohort of animals in which the same neuropathic pain model was induced, but who did either receive no treatment or a sham intervention (eg, electroacupuncture without electrical stimulation). Studies comparing physiotherapy interventions to other substantive control interventions, such as pharmacology were excluded.

#### Outcome Measures

Studies were included if they reported on the effect of the physiotherapy interventions on biomarkers related to neuropathic pain. Studies were not included if they only reported behavioral outcomes. Examples of neuropathic pain biomarkers could include:

1. Immune system: Immune cell markers (eg, CD68, CD3), markers of immune competent cells (eg, OX-42, GFAP), cytokines/chemokines
2. Neurotrohpins (eg, NGF)
3. Opioid system: Neuropeptides (eg, ⍰-endorphine) and receptors (eg, MOR)
4. Neurotrasnmitters (eg, substance P)
5. Ion channels (eg, TRPV1, TRPV8)

### Study Selection

Before carrying out the article selection procedure, a search for duplicates was carried out with MENDELEY. In a first phase, 2 independent reviewers (L.M and A.A.) assessed the eligibility of the studies based on information from title, abstract and keywords. During the second phase, the full text was independently reviewed by both reviewers for eligibility. A third reviewer (C.G.) acted as a mediator when there were differences of opinion between the 2 reviewers, with the 3 reviewers reaching consensus.^25^

### Data Extraction and Management

Data of included studies were extracted by 2 independent reviewers (L.M and A.A.). This involved registered bibliographic data, such as first author and year of publication, animal characteristics (species, age, weight, and gender), neuropathic pain model, treatment groups and intervention characteristics (physiotherapeutic intervention, timing of intervention, number of treatment sessions, duration, dose and location). We also extracted the type of biomarkers including in which tissue they were measured. We attempted to extract means, standard deviations, and *P* values for all biomakers. If available, we recorded behavioral test outcomes to confirm the presence of neuropathic pain. Finally, both authors reached consensus on each item of extracted data. In case of disagreement between the authors, a third author (C.G.) made the final decision.

### Methodological Quality Assessment

#### Risk of Bias Assessment

The risk of bias of each study was assessed using SYRCLE’s risk of bias tool ^26^ scored by 2 independent reviewers (Y.G and E.C.). The tool provides 10 items. These categories are related to selection bias, performance bias, detection bias, attrition bias, information bias, and other biases. Half of these items match those in the tool developed by Cochrane. If there was any disagreement or discrepancy, it was resolved by a third reviewer (J.F.C.). As the tool does not include a specific cut-off, we considered studies to have low risk of bias if they were rated as high bias on less than half of the scoring criteria (<5 out of 10).

#### Reporting Quality

To evaluate the reporting quality of the studies we used the “Animals in research: reporting in vivo experiments” (ARRIVE) guidelines.^27^ The scale has 20 items. Each item refers to a specific section of an article (eg, title, abstract), and other items refer to specific elements of preclinical research (eg, allocation of the animals, housing and husbandry). The score was assessed by 2 independent reviewers (Y.G and E. C.). Any discrepancies were resolved by consensus with a third reviewer (F.C.M). Each ARRIVE item was graduated into 3 descriptive levels: complete (green) when all sub-items in the topic have been described; partial (yellow) when one or more of the sub-items have been described; and incomplete (red) when none of the sub-items have been described. As the tool does not include a cut-off, we considered articles to have good reporting quality if they reported at least 60% of items completely.

#### Qualitative Analysis

For the description of the results, the studies were grouped by type of intervention (eg, exercise, electroacupuncture) as well as type and location of reported biomarkers.

Due to the heterogeneity of reported biomarkers, anatomical measurement sites and measurement methods (eg, gene expression, immunohistochemistry, protein level), and the missing summary statistics in many studies, a meta-analysis could not be carried out.

Instead, we report these findings with heat maps for each intervention and at each location (eg, spinal cord, dorsal root ganglia): color coding was assigned according to the frequency of studies reporting any change on individual biomarker expression (eg, increase, decrease or no change) after the intervention.

## Results

### Selection of the Studies

**Figure 1:**
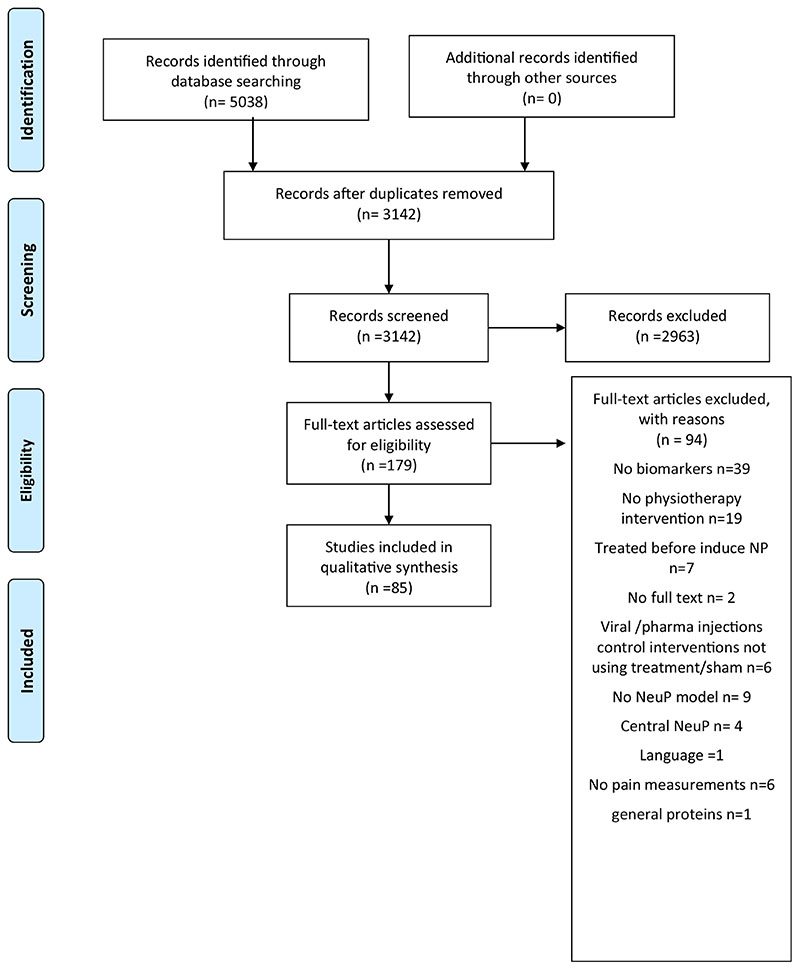
Study flow chart.

The database search retrieved a total of 5,038 articles. After reviewing the titles and abstracts, 179 studies were assessed for eligibility. Of those, 94 were excluded because they did not satisfy the eligibility criteria. This resulted in the inclusion of 85 full-text articles. The flow diagram is shown in Fig 1. The country that produced the most eligible studies is China (38.8%), followed by Brazil (20%) and Taiwan (16.4%). Italy, the United States and Japan contributed with 4.7% each, while Spain, South Korea and Turkey produced 3.5% of included studies. After the selection process, all articles were written in English. No articles in Spanish were found.

### Risk of Bias Analysis

Only 2 of the 85 papers had a low risk of bias, obtaining a 5 per 10 score on the SYRCLE tool. The remaining articles had a high risk of bias (Table 1).

### Reporting Quality According to ARRIVE

Fifty-eight (71.6%) out of 85 articles were rated as 60% or more “complete” according to the ARRIVE guidelines. Twenty-one (80.8%) of the 26 articles exploring the effect of exercise are of good quality. Thirty-three percent (1 out of 3) of the acupuncture and joint mobilization articles have low quality. Of the reports on electroacupuncture, 24.14% (7 of the 29) have low methodological quality. All articles on neural mobilization showed good methodological quality (5 out of 5). Of studies including physical agents, 57.9 % (11 out of 19) were of good quality (Supplementary Table 1).

### Characteristics of the Studies

Characteristics of the included articles, such as details of animal species, neuropathic pain models and treatment groups and interventions are shown in Supplementary Table 2.

Most studies reported on electroacupuncture (34.1%) and exercise (30.5%) followed by physical agents (23.5%), neural mobilization (6.2%), and acupuncture and joint mobilization (2.5%).

The most widely used model of neuropathic pain was traumatic nerve injury (78.9%), with chronic constriction injury being the most studied model (55.8%) followed by sciatic nerve cut (13%). Other models reported were diabetic neuropathy, complex regional pain and chemotherapy induced neuropathy. 82.72% of the articles confirmed the presence of NeuP with behavioral tests before treatment started.

Rats were the most prevalent species studied (85.2%) followed by mice (14.8%). Only 1 report with rabbits was included. Whereas 92.5% of studies included only male animals, 7.4 % of studies studied female animals. None of the studies included both sexes.

### Biomarkers Type and Site Examined

The main biomarkers reported are related to the immune system (67.9%) followed by neurotrophins (27.2%), neurotransmitters (16%) and opioid pathways (7.4%. The anatomical sites where the biomarkers were measured included spinal cord (53.0% of studies), followed by the peripheral nerve and dorsal root ganglia (both 30.9%), the brain (13.6%) and blood (4.9%) (Table 2).

## Qualitative Analysis

Supplementary Table 1 contain heat maps reflecting the frequency of studies showing specific directions of effects (up vs downregulation vs no change) of each physiotherapy intervention on biomarkers of neuropathic pain.

### Exercise

Two types of exercises were investigated in the studies, swimming, and treadmill running.

Swimming was one of the two activities studied by 4 out of 26 studies (15.4%). The dose for swimming exercise ranged from 40 to 60 minutes and was performed on 5 days per week. Swimming reduced the concentration of proinflammatory cytokines in the injured nerve tissue,^28^ as well as the concentration of neurotrophins in spinal cord, dorsal root ganglia, and peripheral nerve tissue in the medium term.^29,30^ Only 1 article found no post-treatment differences in BDFN concentrations.^31^ One paper found an increase of GAP-43 in the peripheral nerve.^31^

Treadmill aerobic training was the most used by the studies (23 out of 26 studies, 88.5%), both in isolation and using it against other therapies. The dose of treadmill running ranged from 60 minutes to exhaustion and was performed between 3 and 5 days per week over a period of 3 to 8 weeks. Treadmill running was able to reduce proinflammatory cytokines and increase anti-inflammatory cytokines mainly in peripheral nerves,^32–35^ with changes in DRG and spinal cord also reported.^36–39,33,40,41^ Only one article found increased proinflammatory cytokines in nerve and dorsal horn of the spinal cord.^39^ Only 1 study found no difference in the sub-group “other inflammatory markers” of the immune system^42^ The concentration of neurotrophins was lowered after treadmill exercise.^9,43,44,30^ One study reported increased expression of at least one of these biomarkers when treadmill running was combined with electrical stimulation.^9^ Treadmill running was also effective in reducing the activation of glial cells in DRG and spinal cord.^39,45,46,42,43^ Only 1 article did not find changes in the spinal cord after intervention.^47^ In that experiment, the animals ran until exhaustion,^47^ while in the others it was of a fixed duration.^39,45,47,42,43^ Studies reported a direct relationship between increased expression of inhibitory neurotransmitters, such as serotonin in the brain and spinal cord and exposure to treadmill running.^48,49,44^ Only 1 study found a decrease in neurotrophin expression in the peripheral nerve.^32^ In contrast, the effect on excitatory neurotransmitters was only evaluated in 2 articles, with mixed results, however different neurotransmitters were measured (GABA and Substance P).^50,51^ Two articles reported a decline in the expression of inflammatory markers in the dorsal horn.^47,41^

### Neural Mobilization

Five articles studied neural mobilization. The most frequently reported dose was 20 oscillations per minute for 2 minutes and 25 seconds of rest, for 10 minutes for a total of 10 sessions. Only 1 showed no difference in posttreatment biomarkers of neuropathic pain.^52^ Whereas Giardini et al^52^ evaluated changes in the thalamus, midbrain and PAG, the other studies examined biomarkers in SCDH, DRG, and sciatic nerve. Neural mobilization consistently reduced the concentration of neurotrophic factors and the expression of substance P, TRPV1, and MOR^53,54^ in the spinal cord. One article reported an increased concentration of NGF in the sciatic nerve.^55^ Whereas most studies used the chronic constriction model, one used a diabetic neuropathy model^56^ and reported a decrease in intraneural proin-flammatory cytokines on the treated side.

### Joint Mobilization

Two studies evaluated the effect of joint mobilization on biomarkers of neuropathic pain. The dose for joint mobilization ranged from 1 series of 10 repetitions to 3 minutes series with 30 seconds’ rest. The frequency ranged from every 2 days to 5 consecutive days for a total of 12 to 15 days. Joint mobilization consistently reduced activation of the immune system (glial cells mainly) in the SCDH.^57^ Their effect on cytokine expression revealed controversial results; while the concentration of cytokines in the DRG remained the same after treatment, only anti-inflammatory cytokines increased their expression in the spinal cord.^58^ One of the 2 studies used rhythmic mobilization techniques^57^ and the other high-speed manipulations.^58^ The place of application was different as well as the dose, so the results must be interpreted with caution.

### Physical Agents

Nineteen studies investigated a range of physical agents including laser, therapeutic ultrasound, and transcranial direct current stimulation. The dose for ultrasound most frequently reported was 1 MHz 0.5 to 1 w/cm^2^ during 5 minutes.

Therapeutic ultrasound reduced the expression of substance P in both studies.^59,60^ Further, a reduction of cytokines (tumor necrosis factor [TNF] and interleukin-6 [IL-6])^59^ and TRPV1 expression^60^ was apparent at sciatic nerve and dorsal root ganglia respectively.

Of the 5 articles including laser therapy, only 1 measured the changes generated on enkephalines^61^ with no changes after treatment. Three papers report a decrease of cytokine concentration.^62,63^ All laser treatments increased the concentration of NGF in the sciatic nerve regardless of the time of intervention or parameters applied.^64,63^ Cidral et al^62^ found a decrease in the concentration of TNF but not IL-1 *β* in the SC and the sciatic nerve while Hsieh et al^65^ reported a decrease of several cytokines measured in the sciatic nerve. This difference could be due to the different intensities applied in the studies. Cidral et al^45^ used 80 mW/cm^2^ and 2.5 J/cm^2^ versus 30 mW/cm^2^ and 9 J/cm^2^ used by Hsieh et al^63^ in both studies.

Two studies investigated tDCS. tDCS increased TNF-a concentrations in the brain and spinal cord, whereas IL-1b and IL-10 only changed significantly in the spinal cord, with a decreasing concentration of both cytokines.^66^ tDCS also reduced the activation of glial cells in spinal cord dorsal horn^67^ and decreased BDNF concentrations both in the central nervous system and in blood serum.^68^

Three studies reported on the effect of TENS therapy. TENS could not reduce proinflammatory cytokines (TNF-a) in the sciatic nerve,^69^ in fact 1 study reported an increase in that biomarker.^70^ However, TENS did reduce the concentration of proinflammatory cytokines in the spinal cord.^71^ The glial activity in the spinal cord was reduced after the application of TENS, and the expression of opioid receptors increased in the same location.^71^ Contradictory results were reported regarding the presence of excitatory neurotransmitters in the spinal cord.^72^

The pulse electromagnetic field was consistent in modulating the cytokine concentrations, in both the spinal cord and the peripheral nerve tissue that caused the injury.^73,74^

### Electro-Acupuncture

Electroacupuncture reduced the concentrations of proinflammatory cykines. The doses reported ranged from 1 to 2 mA, fluctuating between 2 and 100 Hz, 1.05 to 2.85 milli seconds for 30 minutes. Most of the changes seem to occur in the dorsal horn^75-80^ although changes in the nerve,^81,82^ blood,^83^ and DRG^84^ were also reported. In contrast, four articles did not find changes in cytokine concentrations following electroacupuncture.^81,83,85,76^

The effect of electroacupuncture reported on neurotrophins has been mixed. Articles reported decreased concentrations of nerve growth factors (NGF and BDNF) in dorsal root ganglia and spinal cord dorsal horn^86,87,76,88^ while others obtained significant increases in the same anatomical sites for NGF,^84^ BDNF,^89^ and GDNF.^90^ These differences may be due to the starting times and duration of treatment. It seems that most of the articles that reported a decreased concentration^86,87,76,88^ had a treatment duration greater or equal to 2 weeks. In contrast those that increased pain markers expression only treated the animals for 1 week.^89,84^

### Acupuncture

The three acupuncture articles included were very heterogeneous. Wang et al^91^ and Tang et al^92^ found a significant decrease in the concentrations of cytokines. Tang et al does not report the first day of intervention. While Wang et al performed the treatment 1 day after surgery and for a period of 14 days,^91^ Chang et al started the intervention 24 days after surgery, during a period of 5 days.^93^ The location of biomarker measurement were different; Wang et al measured cytokines in the blood meanwhile Tang et al measured in the sciatic nerve, Chang et al measured Cdc2 and P-vim in the sciatic nerve and DRG with no difference after treatment.^93^ Tang performed the treatment for 20 minutes in contrast to the others two articles, that did the same 30-minute daily dose was applied, but the duration of treatment varied between 1 and 2 weeks.

## Discussion

This systematic review summarizes the results of 85 studies that report the influence of different types of physiotherapy modalities on biomarkers of peripheral neuropathic pain in pre-clinical models. The 2 most studied interventions were electro-acupuncture and exercise, with neural mobilization, joint mobilization and physical agents being less commonly studied. The most frequently measured biomarker group was related to the neuro-immune system, specifically cytokines. The dorsal horn is the anatomical site where biomarkers were measured most frequently. Most studies, despite their heterogeneous nature, report significant postintervention changes of the biomarkers of neuropathic pain. Our findings indicate that physiotherapy interventions downregulate the expression of pronociceptive (eg. immune system or neurotrophins) markers and upregulate the expression of markers that dampen neuropathic pain (eg. opioid system). However, risk of bias was high in 97.5% of studies.

Our findings about the most common model is similar to previous reviews about preclinical models of NP were traumatic injury (78.9%) is the most commun.^94^ Although neuropathic pain induced by chemotherapy^95^ or diabetic painful neuropathy are growing problems,^96^ the models of neuropathic pain induced by chemotherapy and diabetic neuropathy have not been used very often in preclinical physiotherapy studies (2.5% and 11.1%, respectively).

### Effects of Physiotherapy

Exercise was one of the main interventions studied, specifically swimming and running (treadmill). It is well established that aerobic exercise induces analgesic effects in preclinical models.^97^ Our results demonstrate that aerobic exercise has promising effects on biomarker modulation in neuropathic pain. There seems to be a consistent effect of aerobic exercise on the modulation of markers of neuro-inflammation in the peripheral and central nervous system. Other biomarkers, such as neurotrophins and neurotransmitters are also modulated by exercise. Of note, studies which did not demonstrate an effect on biomarkers used exercise duration of less than 40 minutes,^29,31^ perhaps insufficient time to generate changes. In contrast, studies showing an effect on biomarkers included sessions with a duration between 60 and 90 minutes.^28,30^ For treadmill running, only 1 article did not find changes after intervention.^46^ In this experiment the animals ran until exhaustion,^46^ while in the others it was of a fixed duration.^39,45,46,42,43^ It could thus be speculated that reaching exhaustion may counteract the positive effects of physical activity in regulating glial cell activity.

Neural Mobilizations have shown efficacy in human trials of patients with referred leg or arm pain of neural origin,^98^ however their exact mechanisms of action remain speculative. In line with findings in animal models,^54,56^ neural mobilizations improve mechanical hyperalgesia in patients after neural mobilization intervention.^99^ Our findings indicate that neural mobilizations may exert their beneficial effect through modulating neuroinflammation, opioid system, and neurotrophins. The ability of neural mobilization to disperse fluids has been reported with cadaveric models.^100^ In patients, there is also some indication that neuroinflammation may be a target. Schmid et al reported a reduction of intreanueral edema after 1 week of neural mobilization in patients with carpal tunnel syndrome.^101^

Although Joint mobilization techniques are often used, they seem to have only short term analgesic effects in humans.^102,103^ In addition they are not usually used for neuropathic pain, but for nociceptive pain.^104,105^ Both preclinical studies included in our systematic review reported a decrease of mechanical hyperalgesia after the interventions.^57,58^ Similarly, Krouwel et al reported an increase on the pain pressure thresholds in humans after a lumbar joint mobilization.^106,103^ Interestingly, our data indicate that joint mobilization may exert their beneficial effects through modulation of glial cells and cytokines. However, only two articles were included, both using different techniques which make it difficult to draw firm conclusions.

Physical agents are often used clinically as analgesic treatments. However, their clinical benefit remains contradictory. For instance, a Cochrane review about the use of TENS in adults with neuropathic pain could not draw firm conclusions whether TENS is effective for pain control due to the very low quality of the evidence.^107^ Another review from Akyuz et al conclude that physical modalities such as ultrasound or laser are not effective for the treatment of neuropathic pain when applied alone.^108^ Our data suggest that physical agents mainly seems to modulate neuropathic pain through regulation of neuroinflammation, such as a downregulation of TNF and IL-1*β* which are associated with the maintenance of neuropathic pain after peripheral injury.^109^ Nevertheless, physical agents could also modulate other biomarkers, for instance neurotrophins or neurotransmitters.

Electroacupuncture has shown some evidence in reducing pain in patients with osteoarthritis mediated by *β* -endorphins.^110^ Human evidence for the effect of electroacupuncture on neuropathic pain remains controversial. Penza et al did not find pain improvements following electroacupuncture treatment in patients with neuropathic pain^111^ whereas Galantino et al reported some improvement in patients with human immunodeficiency virus-related peripheral neuropathy.^112^ In both reports the number of patients included was small, so these results remain preliminary. Our findings indicate that electroacupuncture may exert beneficial effects through modulating neuroinflammation, regulating neurotrophins and neurotransmitters as well as decreasing ATP and ion channels, such as TRPV1.^113-115, 85,76,116,84, 117, 79,118^ Another possible mechanism is that this type of electrical stimulation may be activating the endogenous opioid system by the release of enkephalins and b-endorphins.^119^

As we only identified three articles about acupuncture, it is difficult to hypothesize about its mechanisms of action. Preliminary data suggest that similar to electro-acupuncture this technique might modulate the activation of the neuro-immune system,^93,92,91^ but further research is needed. In line with our preclinical findings, a Cochrane review about the use of acupuncture in humans with any type of neuropathic pain reports limited evidence.^120^ Another review about acupuncture and its effect on pain could also not establish a clear relationship between the technique and the analgesics effects in humans.^121^

### Implications for Humans

The importance of specific biomarkers to maintain neuropathic pain is not only clear in preclinical models,^122^ but also in humans.^123^ Our findings suggest that Physiotherapy can modulate biomarkers related to neuropathic pain in preclinical models. Although the most studied biomarkers related to the immune system and neurotrophins, this review identified other targets, such as neurotransmitters or the opioid system. In recent years, several publications have reported the possible relationship between the presence of neuropathic pain and some of the reported biomarkers of humans. For instance, neuroinflammation is thought to play a crucial role in the generation and maintenance of neuropathic pain in preclinical models^124^ Similarly, there is a growing body of evidence confirming the importance of neuro-inflammation in neuropathic pain in humans. Inflammation in the pathophysiology of neuropathic pain^123^ This is apparent both in patients with focal nerve injuries,^65^ but also in patients with polyneuropathies.^125,126^ As such, our findings indicate that physiotherapy can modulate biomarkers that are relevant in patients with neuropathic pain.

In addition to the neuroimmune system, other systems may influence the presence of NP. For example, neurotrophins have been implicated with neuropathic pain. For Instance, NGF acts as a pathogenic pain mediator^127^ and also in humans, high levels of NGF have been associated with pain.^128^ BDNF shows similar hyperalgesic effects and its presence in the dorsal root ganglia and the spinal cord correlate with neuropathic pain behaviour.^129^ The dysfunction of the opioid system has been described in preclinical^130^ and in humans with NP.^131^ And other indirect measure from the opioid system is the conditioned pain modulation which is mediated by the endogenous opioid system.^132^ This type of alteration has been reported in patients with different types of NP, such as complex regional pain syndrome^133^ or carpal tunnel syndrome.^134^ These 2 systems look like a promising target which required further investigation in human trials.

So far, pharmacological management has been the first line of treatment for NP in humans. Tricyclic antidepressants (eg, amitriptyline), and serotoninnoradrenaline reuptake inhibitors (eg, duloxetine) or anticonvulsants (eg, pregabalin) have been use as first line option.^4^ Also opioids, like tramadol have been use to target the opioid system.^5^ Even Combination therapy have been used in these kind of patients, for instance the use mixed of morphine and gabapentin provided better pain relief together but that gain was also modest.^135^ Despite of this evidence, some trials have report controversial results^136,137^ in addition of the concerns about side effects reported of long term used^138^ advises on looking for new, safer treatment options.

Future targets to investigate are the endogenous cannabinoids, such as CB2 receptor which recently have been shown to increase hypersensitivity in models of neuropathic pain^74^ and we have not found this to have been evaluated in physiotherapy studies.

Whereas the results of this study seem to suggest promising effects of biomarker modulation of physiotherapy interventions for peripheral neuropathic pain, these findings cannot be directly translated to understand the mechanism of these therapies in humans. Nevertheless, these findings can provide guidance on the type and design of future physiotherapy interventions in clinical trials.

One of the most recommended treatment option for the treatment of neuropathic pain, a part of pharmacology, is exercise.^18,19^ In humans is well establish that the hypoalgesic effects are correlated with the intensity or the prescribed dose.^139-141^ Only three articles analyzed in this review reported the intensity of the intervention.^37,38,39^ The 3 reports used low intensity prescription and they reported changes in biomarkers concentrations in both, locally and remotely. This is intriguing since, in humans, has been reported central activation mechanisms only with high intensity.^141^ Future research taking the intensity into account should be done.

### Limitations

We have identified some limitations in our review. As we have not extracted the data from behavioral assessments, we cannot classify the interventions and the posterior analysis by the potential neuropathic pain mechanisms. Only studies written in English were included after the selection process. The heterogeneity of the measurement methods as well as the large number of different biomarkers analyzed challenges the interpretation. Of note, 92.5% of studies only included male rats. It is well established that pain behavior and underlying mechanisms differ according to sex,^142^ thus limiting the generalizability of our findings. Importantly, risk of bias was high and reporting according to the ARRIVE guidelines was poor in the majority of studies. The inconsistent reporting of summary statistics prevented a meta-analysis. Poor reporting and methodological quality have been identified as major challenges in preclinical research including in the pain field.^143,144^ With the recent publication of the ARRIVE guidelines, it is hoped that the quality of preclinical studies and their reporting will improve, thus facilitating future systematic reviews.^27^

## Conclusion

Our results suggest that exercises, electro-acupuncture, neural mobilization, and physical agents modulate biomarkers of neuropathic pain in preclinical models.

Only few studies were available for joint mobilization and acupuncture, thus preventing firm conclusions. Physiotherapy interventions seem to regulate the expression of a range of biomarkers particularly associated with the neuro-immune system, opioid system, neurotransmitters, neurotrophins, and receptors. The high risk of bias and poor reporting quality however prevents firm conclusions. Nevertheless, our findings may be used to inform the design of future human studies. Future preclinical studies need to follow higher standards of methodological quality and reporting to advance this promising field.

## Supplementary Material

Supplementary Information

## Figures and Tables

**Table 1:** Risk of Bias Assessment Using the SYRCLE Tool

-	Selection bias	Selection bias	Selection bias	Performance bias	Performance bias	Detection bias	Detection bias	Attrition bias	Reporting bias	Other
**Reference**	Was the allocation sequence adequately generated and applied?	Were the groups similar at baseline or were they adjusted for confounders in the analysis?	Was the allocation adequately concealed?	Were the animals randomly housed during the experiment?	Were the caregivers and/or investigators blinded from knowledge which intervention each animal received during the experiment?	Were animals selected at random for outcome assessment?	Was the outcome assessor blinded?	Was incomplete outcome data adequately addressed? of selective outcome reporting?	Are reports of the study free of selective outcome reporting?	Was the study apparently free of other problems that could result in high risk of bias?
**Almeida, 2015**										
**Belmonte, 2018**										
**Bobinsky, 2011**										
**Bobinsky, 2015**										
**Bobinsky, 2018**										
**Cha, 2010**										
**Cha, 2012**										
**Chang, 2013**										
**Y-W. Chen, 2013**										
**Y-W. Chen, 2015 (US)**										
**Chen, 2015 (TT)**										
**Y-W. Chen, 2012**										
**Chen, 2015**										
**Cidral, 2013**										
**Cioato, 2016**										
**Coradini, 2015**										
**Cobinachi, 2010.**										
Cobianchi, 2013*										
**Dong, 2005 (Samatostatine)**										
**Dong, 2005**										
**Filho, 2016**										
**Giardini, 2017**										
**Giuliani, 2004**										
**Gong, 2017**										
**Hsieh, 2012**										
**Hsieh, 2017**										
**Huang, 2017**										
**Hung, 2014**										
**Kami, 2016a**										
**Kami, 2016b (Jpain)**										
**Korb 2010**										
**Li, 2019**										
**Liang, 2015**										
**Liang, 2016**										
**Lin, 2015**										
**Liu, 2017**										
**Liu, 2015**										
**López-Álvarez, 2015.**										
**López-Álvarez, 2018.**										
**Ma, 2018.**										
**Manni, 2011.**										
**Martins, 2011**										
**Martins, 2017.**										
**Matsuo, 2014.**										
**Mert, 2015 (a).**										
**Mert, 2015 (b).**										
**Mert, 2017**										
**Nori, 2013.**										
**Santos, 2012**										
**Santos, 2018**										
**Shao, 2015**										
**Shi, 2013**										
**da Silva, 2015**										
**da Silva Oliveira, 2018**										
**Somers, 2003**										
**Somers, 2009**										
**Song, 2016**										
**Su, 2018**										
**Sumizono, 2018**										
**Sun, 2004**										
**Tang, 2020**										
**Thakur, 2016**										
**Tian, 2018**										
**Tsai, 2017**										
**Tu, 2015**										
**Tu, 2018**										
**Wang, 2009**										
**Wang, 2014**										
**Wang, 2016 (a)**										
**Wang, 2016**										
**Wang, 2018**										
**Wang, 2020**										
**Wang, 2021**										
**Xia, 2019**										
**Xu, 2016**										
**Xue, 2015**										
**Yang, 2018**										
**Yong-Hui, 2014**										
**Yu, 2013**										
**Yueh-Ling, 2012**										
**Zhang, 2014**										
**Zhang, 2018**										
**Zhao, 2020**										
**Zhu, 2017**										

Green: low risk of bias; yellow: unclear risk of bias; red: high risk of bias.

**Table 2:** Characteristics and Findings of the Included Studies in Relation to Biomarkers

Reference	Groups	Anatomical Level	Biomarkers	Main Results	P value
Chang, 2013	NC NC + acupuncture	POD 7 Sciatic nerve DRG Sciatic Nerve	NF-200-stained axons (Quantification of axonal regeneration) % number of labelled neurons Quantification of Hoechst-stained nuclei Cdc2 P-vim	Increased by acupuncture No difference ? ?	*P* < .05
Wang, 2009 Tang, 2020	CCI CCI + acupuncture Control Diabetic neuropathy Diabetic neuropathy + acupuncture	POD 15 blood Serum spinal cords	IL-1B CXCR3 TNF-*α* IL-1 *β* IL-6 P2×4	Decrease CCI+acupuncture Decreased Decreased Decreased Decreased Decreased	*P* < .01 P < .001 P < .001 P < .001 P < .001 P < .001
Cha, 2010	NT NT + EA	POD ? Spinal cord	Neuronal nitric oxide synthase-positive neurons	Decrease by EA in Rexed area I–II but no difference in Rexed area III–V and X	*P* < .05
Cha, 2012	NT NT + EA	POD? Peripheral nerves DRG	IL-1b IL-6 TNF-Alfa IL-1beta IL-6 TNF-Alfa	Decrease by EA Decrease by EA Decrease by EA Decrease by EA No difference No difference	*P* < .05 *P* < .05 *P* < .05 *P* < .05. - -
X.-M. Chen, 2015	CCI CCI + EA	POD 14 Spinal cord	P2×4R IFN-g	Decrease by EA Decrease by EA	*P* < .01 *P* < .01
Dong, 2005 (a)	CCI CCI + EA	POD 14, 21 and 28 DRG Spinal cord	GDNF (WB) GDNF (IR) GDNF (PCR) GFR*α*-1 (WB) GFR*α*-1 (PCR) GDNF (IR)	Increase by EA at day 14 Increase by EA at days 21, and 28 Increase by EA at day 21 Increase by EA at day 28 Increase by EA at days 14 and 21 Increase by EA at day 28 Increase by EA at day 14 Increase by EA at days 21 and 28 Increase by EA at days 14 and 21 Increase by EA at day 28 Increase by EA at days 14 and 21 Increase by EA at day 28	*P* < .05 *P* < .01 *P* < .05 *P* < .01 *P* < .01 *P* < .001 *P* < .05 *P* < .01 *P* < .05 *P* < .01 *P* < .01 *P* < .001
Dong, 2005 (b)	CCI CCI+EA	POD 14, 21 and 28 DRG Spinal cord	SOM (IR) SOM (PCR) SOM (IR)	Increase by EAat days 14, 21 and 28 Increase by EA at days 14 and 21 Increase by EA at day 28 Increase by EA at day 14 Increase by EA at days 21 and 28	*P* < .01 *P* < .01 *P* < .001 *P* < .05 *P* < .01
Liang, 2016	CCI CCI + EA CCI + sham EA	POD After 73 hours Laminae I-II of ipsilateral Spinal cord dorsal horn (SCDH)	p-p38 MAPK OX–42	Decreased by EA Decreased by EA Decreased by EA	*P* < .01 *P* < .05 *P* < .01
Liu, 2019	CCI CCI + EA	POD 8 Spinal cord	TNF-a IL-1B IL-6 CX3CR1	Decreased by EA Decreased by EA Decreased by EA Decreased by EA	*P* < .01 *P* < .001 *P* < .001 *P* < .001
Shao, 2015	CCI EA strong manual acupuncture (smA) mild manual acupuncture (MA)	POD ? Spinal cord Brain (anterior cingulate cortex)	p-ERK GFAP p-ERK OX42	Decrease (smA= MA)	*P* <.01 smA = MA
Sun, 2004	CCI + PESCCI + needling	POD 48 L5 spinal superficial laminae I-II	NMDA(NR1)	Decrease PES group	*P* < .001
Tu, 2015	CCI CCI + EA	POD 14 ipsilateral L4-6 DRGs L4-L5 lumbar spinal cords, dorsal horn	NT-3 NT-3 IL-1 b GFAP OX-42	Increase EA Increase EA Decrease EA Decrease EA Decrease EA Decrease EA	*P* < .001 *P* < .001 *P* < .001 *P* = .001 *P* = .003
Tu, 2018	CCI CCI + EA	POD 14 Spinal Cord L4-L6	BDNF TrkB	Decrease EA Decrease EA	*P* < .001 *P* < .001
Wang, 2014	CCI CCI + contralateral EA CCI + ipsilateral EA	POD 14 L4-L6 Dorsal Root Ganglia ipsilateral contralateral (P2×3)	ATP ATP	Decrease EA Decrease EA	*P* < .001 *P* < .001
Wang, 2016	CCI CCI + sham EA CCI + EA	POD 14 L4-L5 spinal cord (dorsal horn)	IL-B GFAP TNF-a IL-6 BDNF NGF NT3 NT4	Decrease EA decrease EA EA no difference decrease EA decrease EA decrease EA decrease EA decrease EA	*P* < .05 *P* < .05 *P* < .05 *P* < .05 *P* < .05 *P* < .05 *P* < .05 *P* < .05
Wang, 2018	CCI CCI + EA	POD21 Spinal Cord L4-L6	a7nAChRIL-1B	Increase EA decrease EA	*P* < .01 *P* <.001
Xia, 2019	CCI CCI + EA	POD21 L4-L6.	HMGB1 TLR4 CD1 MyD88 NF-kB	Decrease EA Decrease EA Suppressed EA Suppressed EA Inhibited EA	*P*< .01 *P*< .001 *P*< .01 *P*< .05 *P*< .05
Xu, 2016	CCI CCI + EA	POD 14 L4-L5 Spinal cord ipsilateral	P2×7RIL-1B, IL-18	Decrease EA Decrease EA Decrease EA	*P* < .0001 *P* = .0026 *P* = .0023
Xue, 2015	CCI CCI + EA	POD ? Spinal cord	BDNF P2×4	Increase CCI + EA No significant difference	*P*< .05
Yong-Hui, 2014	CCI CCI + 3 EA CCI + 5EA CCI + 12EA	POD ? Blood Hypothalamus	IL1-B IL-2 IL-12 IL-15 INF-y IL-4-Il-10 TGF-B beta-endorphin beta-endorphin	Decrease 12 EA No significant difference No significant difference CCI No significant difference CCI 12 EA reduce to normal No significant upregulated EA 12 EA upregulated All EA upregulated All EA upregulated	*P*< .05 *P*< .05 *P*< .05 *P*< .05 *P*< .05
Yu, 2013	CCI group CCI + low-frequency EA CCI + high-frequency EA	POD 10 Spinal Cord	P2 × 3 protein P2 × 3 receptor	EA decrease EA decrease	LEA *P* = .045 HEA *P* = .047 Lea versus Hea *P* < .05 to LEA
Zhang, 2014	NT NT + EA	POD 7-28 Brain (arcuate nucleus)	*β*-endorphin	EA increase	*P<* .05
Zhang,2018	CCI CCI + EA	POD 7 L4-L6 spinal cord	GFAP IL-6 TNF-*α* IL-1 *β*	CCI + EAdecrease CCI + EA decrease CCI + EA decrease CCI + EA decrease	*P*< .01 *P*< .01 *P*< .01 *P*< .01
Almeida, 2015	CCI CCI + Swimming CCI + Swimming + Detraining	POD 42 and 70 DRG	BDNF GDNF NGF	Decrease by swimming at day 42; Decrease by swimming + detraining at day 70 No difference Decrease by swimming at day 42; No difference by swimming + detraining	*P*< .05 *P*< .05
Bobinsky, 2011	Non-Exer NC + Exercise-preoperative (Exer 1) NC + Exercise-preoperative-postoperative (Exer 2) NC + Exercise-postoperative (Exer 3)	POD 15 Sciatic nerve Spinal cord	TNF-alfa IL-1beta IL-6R TNF-alfa IL-1beta IL-6R IL-10	Decrease by Exer 2 and Exer 3 Decrease by Exer 1, Exer 2 and Exer 3 No difference No difference Decrease by Exer 2 and Exer 3 Decrease by Exer 1, Exer 2 and Exer 3 Decrease by Exer 1, Exer 2 and Exer 3 No difference	*P<* .05 *P*< .05 *P<* .01 *P*< .05 *P*< .05
Bobinsky, 2015	NC + Sedentary NC + Exercise	POD 15 Brainstem Medullary raphe	5-HT 5-HIAA 5-HT1A 5-HT1B 5-HT2A 5-HT2C 5-HT3A TNF-alfa IL-1beta SERT SERT	Increase by exercise Increase byexercise No difference Increase by exercise Increase by exercise Increase by exercise No difference Decrease by exercise Decrease by exercise Decrease by exercise Decrease by exercise	*P*< .001 *P*< .01 *P*< .05 *P*< .05 *P*< .05 *P*< .05 *P*< .05 *P*< .01 *P*< .05
Bobinsky, 2018	NC + Sedentary NC + Exercise	POD 15 Sciatic nerve Spinal cord	IL-4 IL-1ra IL-5 IL-6 IL-4 IL-1ra IL-5 IL-6 BDNF *β*-NGF GFAP Iba-1	Increase by exercise Increase by exercise No difference No difference Increase by exercise Increase by exercise Increase by exercise No difference Decrease by exercise Decrease by exercise Decrease by exercise bilateral I-II/ipsilat-eral III-VI Decrease by exercise bilateral I-II/ipsilat-eral III-VI	*P*< .05 *P*< .05 *P*< .01 *P*< .01 *P*< .05 *P*< .01 *P*< .001 *P*< .05 *P*< .05 *P*< .01
Y-W. Chen, 2012	CC CCI + Swimming Exercise (CCISE) CCI + Treadmill Exercise (CCITE)	POD 21 Sciatic nerve	Hsp72 TNF-alfa IL-1beta	Increase by CCISE Increase by CCITE ecrease by CCISE and CCITE Decrease by CCISE Decrease by CCITE	*P* < .05 *P* < .01 *P* < .05 *P* < .05 *P* < .01
Cobianchi, 2010.	CCI CCI + EX day3-7CCI + Ex day3-56	POD:7AND 17 Dorsal horn ipsilateral Ventral horn ipsilateral Dorsal horn contralateral Ventral horn contralateral	Cd11bIR GFAP IR	7 d: Decreased by exercise 17 d: Decreased by exercise 7 d: decreased by exercise 17d:No difference	*P* < .01
Cobianchi, 2013	NT NT + TR NT + ES	POD 1, 3, and 8 DRG Spinal cord	NGF NT-3 BDNF GDNF NGF NT-3 BDNF GDNF	Decrease by ES at day3 but not at day 1; No difference at day 8 No difference at day 1, day 3, and day 8 Decrease by ES at day 3 but not at day 1 Decrease by TR at day 8 No difference at day 1 and day 3; Decrease by TR (compared to NT and ES) at day 8 No difference Increase by ES at day 1 but not at day 3; No difference at day 8 No difference at day 1 and day3 Increase by ES at day 8 Decrease by TR (compared to NT+ES) at day8 Decrease by ES+TR (compared to ES) at day 8 No difference Increase by ES+TR (compared to NT and TR) at day 8 Increase by ES+TR (compared to ES) at day 8	*P* < .01 *P* < .05 *P* < .01 *P* < .05 *P* < .01 *P* < .01 *P* < .05 *P* < .001 *P* < .01
Coradini, 2015	CCI CCI + Swim CCI (Obese) CCI + Swim (Obese)	POD? Right median nerve	GAP43 BDNF	Increased by CCI+swim versus CCI No difference between CI+swim (obese) and CCI (obese) No difference between CCI + swim and CCI No difference between CCI + swim (obese) and CCI (obese)	*P* < .05
Gong, 2017	CCI CCI + exercise	POD 31 (Postnatal day 41) Spinal dorsal horn Ipsilateral spinal cord	IL-1B TNF-a CD86 CD68 INOS IL-4 IL-10 CD2016 Arg Ym1 CD206 + Microglia proportion IL-10 (western blot) TNF-a (western blot)	Decreased byexercise Decreased byexercise No difference Decreased by exercise Decreased by exercise Increased by exercise Increased by exercise Increased by exercise Increased by exercise Increased by exercise Increased by exercise Increased by exercise Decreased by exercise	*P* < .05 *P* <. 05 *P* > .05 *P* < .05 *P* < .05 *P* < .05 *P* < .05 *P* < .05 *P* < .05 *P* < .05 *P* < .05 *P* < .05 *P* < .05
Huang, 2017	CCI CCI + TU0 CCI + TU CCI + TE CCI + TU0 + TE CCI + TU + TE	PODs 14 and 28 Sciatic nerve	TNF-a IL-6 IL-10	PODs 14 and 28: Decreased by TU, TE, TU0 + TE, TU + TE POD14: Decreased by TU, TE, TU0 + TE, TU + TE POD28: Decreased by TE, TU0 + TE, TU + TE POD14: Increased byTU, TE, TU0 + TE, TU + TE POD28: No difference	*P* < .05 *P* < .05 *P* < .05 *P* < .05 *P* > .36
Hung, 2014	CCI CCI + TT CCI + TU CCI + TT + TU	POD 14 or 28 Spinal cord	IL-6 IL-10 Iba-1	Decrease by TT,TU and TT+TU at day 14 and 28 No difference at day 14; Increase by TT, TU and TT + TU at day 28 Decrease byTT, TU and TT + TU; Decrease byTT + TU (compared to CCI + TTandCCI+TU)	*P* < .008 *P* < .01 *P* < .01
Hung, 2016	CCI CCI + TU CCI + TT CCI + TT + TU	PODs 14 and 28 Spinal cords (L4 -L5)	IL-6 IL-10 Iba1 IR	PODs 14 and 28: Decreased by TT, TU, TT + TU POD28: Greater decrease with TT + TU compared to TT and TU POD14: No difference POD28: Increased by TT, TU and TT + TU POD28: Greater increase with TT + TU compared to TT and TU PODs 14 and 28: Decreased by TT, TU and TT+TU POD28: Greater decrease with TT + TU compared to TT and TU	*P* < .008 *P* < .05 *P* > .58 *P* < .01 *P* < .05 *P* < .01 *P* < .01
Kami, 2016a	CCI-sedentary CCI + running	POD 7 Lumbar spinal cord (L4-5), superficial dorsal horns	GABA GAD65/67	Increased byrunning Increased byrunning	*P* < .01 *P* < .01
Kami, 2016b	CC_PI-sedentary CCI _P + running	POD 7 Lumbar spinal cord (L4-5), superficial dorsal horns	HDAC1 + nuclei HDAC1+/GFAP+ astrocytes HDAC1+/CD11b+microglia CD11b+ H3K9ace+/CD11b+ microglia CD11b+	Decreased by running No difference Decreased by running No difference Increased by running No difference	*P* < .01 *P* < .01 *P* < .01
Korb 2010	NT + trained NT sedentary	POD 35-36 SC, lumbosacral ventral horn SC, lumbosacral, dorsal horn, superficial laminae Magnus raphe nucleus Dorsal raphe nucleus Soleus muscles	Serotonin (5-HT) immunoreactivity (lumbosacral ventral horn) Serotonin inmunoreactivity (superficial laminae of lumbosacral SC) Serotonin inmunoreactivity (magnus raphe nucleus) Serotonin inmunoreactivity (dorsal raphe nucleus) Citrate synthase enzyme activity (soleus muscle)	Increased bytraining No difference No difference No difference Increases by training	*P* < .05 *P* < .05
López-Álvarez, 2015	CCI + ITR1 CCI + ITR2 CCI	POD 8 and 15 paw skin L3-L5 dorsal root ganglia	NGF ski Western blot of NGF NGF in DRG GAP43 in DRG pNKCC1 NKCC1 pKCC2 KCC2 BDNF L3 BDNF L5 Iba1 l3 Iba1 l5	8 days: Decreased by ITR1 15 days: Decreased by ITR1/ITR2 8 days: Decreased by ITR1 8 days: Decreased by ITR1 8 days: Decreased by ITR1 8 days: Decreased by ITR1 15 days: Decreased by ITR1 8 days: Decreased by ITR1 8 days: Decreased by ITR1 15 days: Decreased by ITR1 15 days: Increased by ITR1 8 days: Decreased by ITR1 15 days: Decreased by ITR1 8 days: Decreased by ITR1 15 days: Decreased by ITR1/ITR2 8 days: Decrease by ITR1 15 days: Decreased by ITR1 8 days: Decreased by ITR1 15 days Decreased by iTR1 15 days Decreased by ITR2	*P* <.05 *P* <.05 *P* < .05 *P* <.05 *P* < .01 *P* < .01 *P* < .001 *P* < .05 *P* < .01 *P* < .05 *P* < .05 *P* < .05 *P* < .01 *P* < .05 *P* < .0001 *P* < .05 *P* < .05 *P* < .01 *P* < .05 *P* < .01
López-Álvarez, 2018	NTR-iTR SNTR-sedentary	POD 14 Spinal Cord DH lamiae I-II. Brain. (periaqueductal grey matter (PAG) the locus coeruleus (LC) the dorsal raphe (DRN) the raphe magnus nucleus (RM)	*α*1A immunoreactivity *α*2A *β*2 receptor 5HT2A	ipsilateral horn: Increased by ITR LC and DRN: Increased by ITR No difference lamina II: increased by ITR the contralateral lamina I: Increased by ITR LC: Increased by ITR lamina II: Increased by ITR Ipsilateral lamina I: Increased by ITR PAG and DRN: Increased by ITR	*P* < .001 *P* < .05 *P* < .001 *P* < .01 *P* < .01 *P* < .01 *P* < .05 *P* < .01
Martins, 2017.	NC NC + eccentric exercise 6 m/min NC + eccentric exercise 10 m/min NC + eccentric exercise 14 m/min	POD 63 sciatic nerve tissues triceps surae	IL-1*β* TNF-*α* IL-4 IL-1Ra IGF-1	No difference Muscle: Decreased by Exercise Nerve: No difference No difference No difference Nerve: Increased by exercise Muscle: no difference	*P* < .03 *P* < .01
Sumizono, 2018	CCI CCI + high-frequency exercise CCI + low-frequency exercise	POD 21 and 35 Dorsal HORN laminae I-III midbrain PAG	BDNF MOR GFAP Iba1 B-endorphin met-enkephalin	Decrease HFE 5 w Decrease all exercise 5 w Decrease all exercise 5 w Decrease all exercise 5 w Increase all exercise 3 w 5 w Increase all exercise 3 w 5 w	*P* < .05 *P* < .05 *P* < .05 *P* < .05 *P* < .05 *P* < .05
Tian, 2018	NT NT + swimming	PODs 21, 42 and 49 SC L4–L6 DRG L4-L5 Tibial nerve (neuroma)	NGF (protein levels, ipsilateral SC) BDNF (protein levels, ipsilateral SC) NGF (protein expression, ipsilateral DRG) BDNF (protein expression, ipsilateral DRG) NGF (protein expression, ipsilateral neuroma) BDNF (protein expression, ipsilateral neuroma)	Day42: Decreased by swimming Day49: Decrease by swimming Day21: No difference Days 42 and 49: Decreased by swimming Day21: No difference Day21: Decreased by swimming Days 42 and 49: No difference Day21: Decreased by swimming Days 42 and 49: No difference Day21: Decreased by swimming Days 42 and 49: No difference Day21: Decreased by swimming Days 42 and 49: No difference	*P* < .01 *P* < .05 *P* < .01 *P* < .05 *P >* .05 *P* < .01 *P >* .05 *P* < .05 *P >* .05 *P* < .01 *P >* 0.05
Tsai, 2017	CCI CCI + 0%-incline treadmill CCI + 8%-incline treadmill	POD 26 sciatic nerve	IL-10 IL-6 TNF-a	Increase 8% treadmill Decrease 8% treadmill Decrease 8% treadmill	*P* < .05 *P* <.01 *P* < .05
Wang, 2016	NC NC + ExNC+EX+EA	POD 31 Tibia	. Substance P	Decrease by exercise and exercise + EA Decrease exercise + EA versus exercise	*P* < .05 *P* < .05
Martins, 2011	NC NC + Anesthesia NC + AJM	POD 35 Spinal cord	GFAP CD11b/c	Decrease by AJM Decrease byAJM (compared to anesthesia) Decrease byAJM Decrease byAJM (compared to anesthesia)	*P* < .01 *P* < .05 *P* < .01 *P* < .05
Song, 2016	CCI de-CCI de-CCI+ASMT	POD 28 Dorsal Root Ganglia neurons L4-L5 Blood Spinal cord L3-L6	c-FOS IL-10 DRG IL-1B, IL-10, Tonfa IL-1B (DRG and SC) TNF-a (DRG and SC) IL-10(SC)	Decrease de-CCD + SMT Suppressed de-CCD + SMT SMT same SMT reduce SMT same SMT increase	*P* <.01 *P* <.01 *P* < .05 *P* < .01
da Silva, 2015	CCI CCI + NM	POD 24 Sciatic nerve	NGF MPZ	Increase by NM Increase by NM	*P* < .01 *P* < .01
Giardini, 2017	CCI CCI + NM	POD ? Thalamus Midbrain VPL and PAG	GFAP OX-42 BDNF GFAP OX-42 BDNF GFAP OX42 BDNF	No difference No difference No difference No difference No difference No difference	*P >* .05 *P >* .05 *P >* .05 *P >* .05 *P >* .05 *P >* .05
Santos, 2012	CCI CCI + NM	POD 24 Dorsal root ganglia Spinal cord	NGF GFAP NGF GFAP	Decrease NM	*P* < .05
Santos, 2018	CCI CCI + NM	POD 24 Dorsal root ganglia L4-L6	Substance P expression of TRPV1 protein expression MOR protein expression DOR protein expression KOR b-actin	Decrease NM Decrease NM Decrease NM Not observe immunoreactivity of these receptors not observe Immunoreactivity of these receptors No differences were observed	*P* < .001 *P* < .001 *P* < .001
Zhu, 2017	diabetes diabetes + neural mobilization	POD 31 Sciatic nerve left (no treatment) Sciatic nerve right (treatment) Dorsal root ganglion	. IL-1B TNF-a IL-1B TNF-a IL-1B TNF-a	No significant different MN decrease versus contralateral side MN decrease versus contralateral side	*P*= .023 *P*= .004
Chen, 2015	CC I CCI + TU-0 CCI + TU-0.25 CCI + TU-0.5 CCI + TU-1	POD 28 sciatic nerve	TNF-a IL-6 NK-1R substance P	TU-1 decrease TU-1 decrease All TU decrease All TU decrease	*P* <.01 *P* <.05 *P* <.05 *P* <.05
Cidral, 2013	NC NC + LEDT	POD 13 Spinal cord Sciatic nerve	TNF-alfa IL-1beta IL-10 TNF-alfa IL-1beta IL-10	Decrease by LEDT No difference No difference Decrease by LEDT No difference No difference	*P* < .05 *P* < .05
Cioato, 2016	CCI CCI + sham tDCS CCI + tDCS	POD 24 and 29 Cortex Spinal cord Brainstem	TNF-alfa IL-1beta IL-10 TNF-alfa IL-1beta IL-10 TNF-alfa IL-1beta IL-10	Increase by tDCS at day 29 but not at 24 No difference No difference Increase by tDCS at day 29 day but not at 24 Decrease by tDCS at days 24 and 29 Decrease by tDCS at day 29 but not at24 No difference No difference No difference	*P* < .05 *P* < .05 *P* < .05 *P* < .05
Filho, 2016	CCI CCI + Sham tDCS CCI + tDCS	POD 24 or 29 Serum Spinal cord Cortex Brainstem	BDNF BDNF BDNF BDNF	Decrease by tDCS at day29 but not at 24 Increase by tDCS at day 29 but not at 24 Decrease by tDCS at day 24 but not at 29 Decrease by tDCS at days 24 and 29	*P* < .05 *P* < .05 *P* < .05 *P* < .05
Giuliani, 2004	CCI CCI + laser	POD? Laminae I and II of the dorsal horn of spinal cord (L3-L5)	Enkephalin mRNA	No difference	
Hsieh, 2012	CCI + laser CCI + sham	POD 14 Sciatic nerve	H&E study (nuclei percentage) ED1 immunoreactivity TNF-a IL-1B Cytokine HIF-1a-positive cells (inmunor-eactivity) HIF-1a (protein levels, immunoblotting) VEGF positive cells (inmunor-eactivity) NGF positive cells (inmunor-eactivity) S100 positive cells (inmunor-eactivity) VEGF (protein levels, immunoblotting) NGF (protein levels, immunoblotting)	Decreased by laser Decreased by laser Decreased by laser Decreased by laser Decreased by laser Decreased by laser Decreased by laser Increased by laser Increased by laser Increased by laser Increased by laser Increased by laser	*P* < .05 *P* < .05 *P* < .05 *P* < .05 *P* <. 0001 *P* = 006 *P* = .006 *P* = .009 *P* = .002 *P* = .005 *P* = .009 *P* = .002
Hsieh, 2017	Oxaliplatin + TUS Oxaliplatin + shamTUS	POD 24 L2–L6 DRG. Superficial laminae (dorsal horn) in lumbar spinal cord (at segments L2 -L6)	TRPM8 TRPV1 SP-like immunoreactivity	Decreased byTUS No difference Decreased byTUS	*P* < .05 *P* > .05 *P* < .05
Lin, 2015	CCI CCI + HFS	POD 7 affected sciatic nerve	TNF-a	No difference	
Liu, 2017	CCI + sham PEMF CCI + PEMF	POD 14 Sciatic nerve Dorsal root ganglion Spinal cord	HCN1 mRNA HCN2 mRNA	No difference No difference	
Matsuo, 2014.	CCI CCI + TENS 1 w CCI + TENS 2 w	POD 8 spinal cord dorsal horn	Iba1 immunoreactivity BrdU-positive/Iba1-positive GFAP immunoreactivity p-p38 in microglia PKC-y p-CREB MAP kinases (p-p38, p-ERK1/ 2, p-JNK) proinflammatory cytokines (IL-1,TNF-, IL-6) opioid receptors (μOR and OR)	Decreased by TENS Decreased by TENS Decreased by TENS Decreased byTENS Decreased by TENS Decreased by TENS Decreased by TENS Decreased by TENS Dncreased by TENS	*P* < .05
Mert, 2015a	sham PMF (SPMF) PMF-AD PMF-AW	POD 28-35 sciatic nerve tissues	IL-1 beta IL-6 IL-10	Decreased by PMF Decreased by PMF increased by PMF PMF-AD > PMF-AW	*P*< .05
Mert, 2017	CCI + PMF CCI + SPMF	POD: 35 sciatic nerve tissues	IL-1b IL-6 IL-10	Decreased by PMF Decreased by PMF Increase by PMF	*P*< .05 *P*< .05 *P*< .05
Somers, 2003	CCI CCI + TENS	POD 12 Spinal cord	Aspartate Glutamate Glycine GABBA	Decrease by TENS Decrease by TENS Decrease by TENS No difference	*P*< .05 *P*< .05 *P*< .05
Somers, 2009	CCI CCI + high frequencyTENS contralateral CCI + low-frequency TENS CCI + randomly TENS	POD ? Dorsal Horn	Aspartate Glutamate Glycine GABA	Increase randomly TENS Increase randomly TENS Increase randomly TENS Increase high frequencyTENS	*P*< .001 *P*< .001 *P*< .001 *P* < .014
Su, 2018	NC NC + High-frequency immediately(HFI) NC + High-frequency 7 days after(HFL) NC + Low-frequency immediately (LFI) NC + Low-frequency 7 days after (HFL)	POD:4 wk after treatments The distal end of the nerve Dorsal root ganglion Somatosensory cortex and hippocampus	S-100 Neurofilament (NF) TNF-a Synaptophysin TNF-a Synaptophysin	Increased by HFI and HFL versus NC and LFI Increased by HFI and HFL versus NC and LFI Increased by HFI versus NC and HFL Increased by HFI versus NC and HFL Increased by HFI versus NC and HFL Increased by HFI versus NC and HFL	*P*< .01 *P*< .01 *P*< .01 *P*< .01 *P*< .01 *P*< .01
Yang, 2018	CCI + sham-rTMS group CCI + 1 Hz group CCI + 20 Hz group	POD 13 L4-L6 Dorsal Root Ganglia ipsilateral Dorsal horn I-IV	nNOs/B-actin GFAP	CCI + 20 HZdecrease 20 HZ CCI + 20 Hz decrease	*P*< .01 *P*< .05
Yueh-Ling, 2012	CCI and treated with laser CCI and treated with sham irradiation	POD sciatic nerve	IL-1B TNF-a HIF-1a VEGF NFG	Decrease after laser Decrease after laser Decreased after laser Increase in laser Increase in laser	*P* < .0001 *P*< .0001 *P* = .006 *P* = .009 *P* = .002
Wang, 2020	Sham Injury + EA Injury	Spinal cord	IRF8 CD11b CX3CRl	Decreased Decreased Decreased	*P*< .001 *P*< .001 *P*< .001
Li, 2019	CIPN CIPN + EA CIPN + sham EA	POD 14 L4–6 DRGs Spinal cord dorsal horn (SCDH)	TRPV1 (normalized fluorescence intensity [%]) TRPV1 (% of TRPV1 + Neuron [among neuron+) TRPV1 (Western blotting) TLR4 MyD88 GFAP (staining intensity) GFAP (number of positive cells) OX42 (staining intensity) OX42 (number of positive cells)	Decreased by EA versus sham EA Decreased by EA versus sham EA Decreased by EA versus sham EA Decreased by EA versus sham EA Decreased by EA versus sham EA Decreased by EA versus sham EA Decreased by EA versus sham EA Decreased by EA versus sham EA Decreased by EA versus sham EA	*P* <.01 *P* <.01 *P* <.01 *P* <.01 *P* <.01 *P* <.01 *P* <.01 *P* <.01 *P* <.01
Hsieh, 2017	Oxaliplatin + TUS Oxaliplatin + shamTUS	POD 24 L2-L6 DRG. Superficial laminae (dorsal horn) in lumbar spinal cord (at segments L2–L6)	TRPM8 TRPV1 SP-like immunoreactivity	Decreased byTUS No difference Decreased byTUS	*P*< .05 *P >* .05 *P*< .05
Zhao, 2020	Control group PTX group PTX + EA group PTX + sham EA group	Spinal cord Serum	GFAP TLR4 NF-κ B IL-1 *β* TNF-*α*	Decreased Decreased Decreased Decreased Decreased	*P*< .05 *P*< .01 *P*< .01 *P*< .01 *P*< .01
Belmonte, 2018	CPIP CPIP + Exercise continous CPIP + Exercise interval protocol	POD 11 Spinal cord	TNF-alfa IL-1beta IL-6 IL- 10 ERK1/2 AKT1/2/3	Decrease by exercise continuous protocol and exercise interval protocol No difference Decrease by exercise continuous protocol and exercise interval protocol Increase by exercise continuous protocol and exercise interval protocol Increase by exercise continuous protocol; decrease by exercise interval protocol No difference	*P* < .05 *P* < .05 *P* < .05 *P* < .05
Manni, 2011.	12 STZ group 12 STZ group + EA	POD 28 skin DRG	NGF skin NGF Spinal Cord substance P (SP) skin substance P (SP) spinal cord NGF receptor TrkA skin pTyr496-TrkA transient receptor potential vanilloid 1 (TRPV1) skin spinal TrkA pTyr496-TrkA in the spinal cord TRPV1 in spinal cord GABA-GAD-67	No difference Decreased by EA Decreased by EA Decreased by EA Decreased by EA Decreased by EA Increased by EA Decreased by EA Decreased by EA Decreased by EA Increased by EA	*P* < .05
Nori, 2013.	DN DN + EA	POD:28 DRG	NGF Protein. NGF mRNA production. NGF Receptor: TrkAmRNA TrkA protein pTyr496-TrkA mRNA-p75NTR p75NTR protein ERK1-2 Akt JNKp38 phospho-IκB-*α* phosphorylation of the IκB-*α* TRPV-1 phosphorylated p38	Decreased by EA No difference Decreased by EA No difference Decreased by EA No difference Decreased by EA No difference No difference Increased by EA Increased by EA Increased by EA Decreased by EA No difference	*P* < .05
Shi, 2013	Diabetes diabetes + EA	POD 30 Dorsal root ganglia L4-L5	. CBS (cystathionine b synthase) p65 b-actin NF-kB	Decrease EA Decrease EA Decrease EA No difference	. *P* < .05. *P* < .05. *P* < .05.
Y-W. Chen, 2013	Sedentary + DN Exercise + DN	POD 14, 28 or 56 Spinal cord Peripheral nerves	Hsp72 TNF-alfa IL-6 Hsp72 TNF-alfa IL-6	Increase by exercise No difference No difference Increase by exercise No difference No difference	*P* < .05 *P* < .05
Y-W. Chen, 2015	Sedentary + DN Exercise + DN	POD 14 and 28 Sciatic nerve	IL-10 IL-6 TNF-*α* MDA	Increase by exercise at days 14 and 28 Decrease by exercise at days 14 and 28 Decrease by exercise at days 14 and 28 Decrease by exercise at day 14 but not 28	*P* < .0051 *P* < .01 *P* < .01 *P* < .01
Ma, 2018.	DN DN + EX	POD 35 DRG	IL-1b IL-6 TNF-a IL1R IL6R TNFR1	Decreased by exercise Decreased by exercise Decreased by exercise Decreased by exercise Decreased by exercise Decreased by exercise	*P* < .05
Thakur, 2016	1diabetes 2diabetic + exercise	POD 42 Spinal cord dorsal horn	IL-1B macrophage (CD11b, CD6) CGRP	Decrease exercise Decrease exercise Preservation exercise	*P* < .05 *P* < .001
Mert, 2015b	STZ-induced diabetic L-PMF-treated diabetic H-PMF-treated diabetic	POD: 35 Spinal cord sciatic nerve tissues	TNF-alpha IL-1 beta IL-6 IL-10	Spinal cord: decreased L-PMF decreased by H-PMF Sciatic nerve: decreased by L-PMF No difference by H-PMF Spinal cord: decreased by L-PMF increased by H-PMF Sciatic nerve: decreased by L-PMF decreased by H-PMF Spinal cord: decreased by L-PMF No difference by H-PMF Sciatic nerve: No difference by L-PMF Increased by H-PMF Spinal cord: increased by L-PMF No difference by H-PMF Sciatic never: No difference by L-PMF decreased by H-PMF	*P* < .05
da Silva Oliveira, 2018	DN + Sham DN + PBM	POD 35 Sciatic nerve	NGF	Increase by PBM	*P* = .0133
Tang, 2020	Control Diabetic neuropathy Diabetic neuropathy + acupuncture	Serum spinal cords	CXCR3 TNF-*α* IL-1 *β* IL-6 P2×4	Decreased Decreased Decreased Decreased Decreased	*P* < .001 *P* < .001 *P* < .001 *P* < .001 *P* < .001 *P* < .01
Wang, 2021	Control Model EA	Sciatic nerve	IL 1b IL 6 TNF-a	Decreased Decreased Decreased	*P* < .01 *P* < .05

Abbreviations: NC, nerve crush; CCI, chronic constriction injury; NT, nerve transection; CPIP, chronic post-ischemia pain; STZ, streptozocin; DN, diabetic neuropathy; SNTR, sciatic nerve transection and repair; POD, post operative day; ?, not reported; ES, electrical stimulation; PES, percutaneus electrical stimulation; HFE, high frequency exercise; PMF, pulse magnetic field; SPMF, sham pulse magnetic field; EX, exercise; EA, electro-acupuncture; AJM, ankle joint mobilization; SMT, spinal manipulative therapy; HFI, high-frequency immediately; HFL, low-frecuency immediately; tDCS, trasncraneal direct current stimulation; DRG, dorsal root ganglia; PAG, periaqueductal grey; SC, spinal cord; SCDH, spinal cord dorsal horn; WB, western blot; PCR, polymerase chain reaction; IL, interleukin; TNF, tumor necrosis factor; TGF, transformin growth factor; MyD-88, myeloid differentiation primary response 88; NGF, nerve growth factor; NT-3, neurotrophin 3; BDNF, brain derived neurotrophic factor; GDNF, glial cell derived neurotrophic factor; GAP-43, growth asociated protein 43; VEGF, vascular endothelial growth factor; GFAP, glial fribillary acidic protein; MDA, mor M-opioid receptor, dor D-opioid receptor, kor k-opioid receptor; TRPV1, transient receptor potential cation channel subfamily V member 1; NMDA, N-nitrosodimethylamine; TRPV8, transient receptor potential cation channel subfamily V member 8; ATP, adenosine triphosphate; OX-42, IFN-y, interferón gamma; NF-kb, nuclear factor-kb; CX3CR1. chemoline receptor 1, cd11b; CD68, cluster of differentiation 68; CD86, cluster of differentiation 86.
