# Supplementary material for: Effect of Physiotherapeutic Interventions on Biomarkers of Neuropathic Pain: A Systematic Review of Preclinical Literature
Source: J Pain. Author manuscript; Available in PMC 2022 Nov 3. (PMC7613788; doi:10.1016/j.jpain.2022.06.007)
Supplement: Supplementary Information [file EMS152413-supplement-Supplementary_Information.zip › 1-s2.0-S1526590022003509-mmc1.docx]

**Supplementary 1.** ARRIVE reporting quality.

| Reference | Title | Abst | Bgrnd | Objetives | Ethical | Study desing | Procedures | Animals | Housing husbandry | Sample | Allocation | outcome | Statistics | Baseline | Numbers analysed | Estimation | Adverse | Interpretation | | Trasnlation | Funding |
| --- | --- | --- | --- | --- | --- | --- | --- | --- | --- | --- | --- | --- | --- | --- | --- | --- | --- | --- | --- | --- | --- |
| Almeida, 2015 |  |  |  |  |  |  |  |  |  |  |  |  |  |  |  |  |  | |  |  |  |
| Belmonte, 2018 |  |  |  |  |  |  |  |  |  |  |  |  |  |  |  |  |  | |  |  |  |
| Bobinsky, 2011 |  |  |  |  |  |  |  |  |  |  |  |  |  |  |  |  |  | |  |  |  |
| Bobinsky, 2015 |  |  |  |  |  |  |  |  |  |  |  |  |  |  |  |  |  | |  |  |  |
| Bobinsky, 2018 |  |  |  |  |  |  |  |  |  |  |  |  |  |  |  |  |  | |  |  |  |
| Cha, 2010 |  |  |  |  |  |  |  |  |  |  |  |  |  |  |  |  |  | |  |  |  |
| Cha, 2012 |  |  |  |  |  |  |  |  |  |  |  |  |  |  |  |  |  | |  |  |  |
| Chang, 2013 |  |  |  |  |  |  |  |  |  |  |  |  |  |  |  |  |  | |  |  |  |
| Y-W. Chen, 2013 |  |  |  |  |  |  |  |  |  |  |  |  |  |  |  |  |  | |  |  |  |
| Y-W. Chen, 2015 (US) |  |  |  |  |  |  |  |  |  |  |  |  |  |  |  |  |  | |  |  |  |
| Y-W. Chen, 2015 (TT) |  |  |  |  |  |  |  |  |  |  |  |  |  |  |  |  |  | |  |  |  |
| Y-W. Chen, 2012 |  |  |  |  |  |  |  |  |  |  |  |  |  |  |  |  |  | |  |  |  |
| X.-M. Chen, 2015 |  |  |  |  |  |  |  |  |  |  |  |  |  |  |  |  |  | |  |  |  |
| Cidral, 2013 |  |  |  |  |  |  |  |  |  |  |  |  |  |  |  |  |  | |  |  |  |
| Cioato, 2016 |  |  |  |  |  |  |  |  |  |  |  |  |  |  |  |  |  | |  |  |  |
| Coradini, 2015 |  |  |  |  |  |  |  |  |  |  |  |  |  |  |  |  |  | |  |  |  |
| Cobianchi, 2010. |  |  |  |  |  |  |  |  |  |  |  |  |  |  |  |  |  | |  |  |  |
| Cobianchi, 2013* |  |  |  |  |  |  |  |  |  |  |  |  |  |  |  |  |  | |  |  |  |
| Dong, 2005 (Somatostatine) |  |  |  |  |  |  |  |  |  |  |  |  |  |  |  |  |  | |  |  |  |
| Dong, 2005 |  |  |  |  |  |  |  |  |  |  |  |  |  |  |  |  |  | |  |  |  |
| Filho, 2016 |  |  |  |  |  |  |  |  |  |  |  |  |  |  |  |  |  | |  |  |  |
| Giardini, 2017 |  |  |  |  |  |  |  |  |  |  |  |  |  |  |  |  |  | |  |  |  |
| Giuliani, 2004 |  |  |  |  |  |  |  |  |  |  |  |  |  |  |  |  |  | |  |  |  |
| Gong, 2017 |  |  |  |  |  |  |  |  |  |  |  |  |  |  |  |  |  | |  |  |  |
| Hsieh,2012 |  |  |  |  |  |  |  |  |  |  |  |  |  |  |  |  |  | |  |  |  |
| Hsieh, 2017 |  |  |  |  |  |  |  |  |  |  |  |  |  |  |  |  |  | |  |  |  |
| Huang, 2017 |  |  |  |  |  |  |  |  |  |  |  |  |  |  |  |  |  | |  |  |  |
| Hung, 2014 |  |  |  |  |  |  |  |  |  |  |  |  |  |  |  |  |  | |  |  |  |
| Kami, 2016ª |  |  |  |  |  |  |  |  |  |  |  |  |  |  |  |  |  | |  |  |  |
| Kami, 2016b (Jpain) |  |  |  |  |  |  |  |  |  |  |  |  |  |  |  |  |  | |  |  |  |
| Korb 2010 |  |  |  |  |  |  |  |  |  |  |  |  |  |  |  |  |  | |  |  |  |
| Li, 2019 |  |  |  |  |  |  |  |  |  |  |  |  |  |  |  |  |  | |  |  |  |
| Liang, 2015 |  |  |  |  |  |  |  |  |  |  |  |  |  |  |  |  |  | |  |  |  |
| Liang, 2016 |  |  |  |  |  |  |  |  |  |  |  |  |  |  |  |  |  | |  |  |  |
| Lin, 2015 |  |  |  |  |  |  |  |  |  |  |  |  |  |  |  |  |  | |  |  |  |
| Liu, 2017 |  |  |  |  |  |  |  |  |  |  |  |  |  |  |  |  |  | |  |  |  |
| Liu, 2019 |  |  |  |  |  |  |  |  |  |  |  |  |  |  |  |  |  | |  |  |  |
| López-Álvarez, 2015. |  |  |  |  |  |  |  |  |  |  |  |  |  |  |  |  |  | |  |  |  |
| López-Álvarez, 2018. |  |  |  |  |  |  |  |  |  |  |  |  |  |  |  |  |  | |  |  |  |
| Ma, 2018. |  |  |  |  |  |  |  |  |  |  |  |  |  |  |  |  |  | |  |  |  |
| Manni, 2011. |  |  |  |  |  |  |  |  |  |  |  |  |  |  |  |  |  | |  |  |  |
| Martins, 2011 |  |  |  |  |  |  |  |  |  |  |  |  |  |  |  |  |  | |  |  |  |
| Martins, 2017. |  |  |  |  |  |  |  |  |  |  |  |  |  |  |  |  |  | |  |  |  |
| Matsuo, 2014. |  |  |  |  |  |  |  |  |  |  |  |  |  |  |  |  |  | |  |  |  |
| Mert, 2015 (a). |  |  |  |  |  |  |  |  |  |  |  |  |  |  |  |  |  | |  |  |  |
| Mert, 2015 (b). |  |  |  |  |  |  |  |  |  |  |  |  |  |  |  |  |  | |  |  |  |
| Mert, 2017 |  |  |  |  |  |  |  |  |  |  |  |  |  |  |  |  |  | |  |  |  |
| Nori, 2013. |  |  |  |  |  |  |  |  |  |  |  |  |  |  |  |  |  | |  |  |  |
| Santos, 2012 |  |  |  |  |  |  |  |  |  |  |  |  |  |  |  |  |  | |  |  |  |
| Santos, 2018 |  |  |  |  |  |  |  |  |  |  |  |  |  |  |  |  |  | |  |  |  |
| Shao, 2015 |  |  |  |  |  |  |  |  |  |  |  |  |  |  |  |  |  | |  |  |  |
| Shi, 2013 |  |  |  |  |  |  |  |  |  |  |  |  |  |  |  |  |  | |  |  |  |
| da Silva, 2015 |  |  |  |  |  |  |  |  |  |  |  |  |  |  |  |  |  | |  |  |  |
| da Silva Oliveira, 2018 |  |  |  |  |  |  |  |  |  |  |  |  |  |  |  |  |  | |  |  |  |
| Somers, 2003 |  |  |  |  |  |  |  |  |  |  |  |  |  |  |  |  |  | |  |  |  |
| Somers, 2009 |  |  |  |  |  |  |  |  |  |  |  |  |  |  |  |  |  | |  |  |  |
| Song, 2016 |  |  |  |  |  |  |  |  |  |  |  |  |  |  |  |  |  | |  |  |  |
| Su, 2018 |  |  |  |  |  |  |  |  |  |  |  |  |  |  |  |  |  | |  |  |  |
| Sumizon, 2018 |  |  |  |  |  |  |  |  |  |  |  |  |  |  |  |  |  | |  |  |  |
| Sun, 2004 |  |  |  |  |  |  |  |  |  |  |  |  |  |  |  |  |  | |  |  |  |
| Tang, 2020 |  |  |  |  |  |  |  |  |  |  |  |  |  |  |  |  |  | |  |  |  |
| Thakur, 2016 |  |  |  |  |  |  |  |  |  |  |  |  |  |  |  |  |  | |  |  |  |
| Tian, 2018 |  |  |  |  |  |  |  |  |  |  |  |  |  |  |  |  |  | |  |  |  |
| Tsai, 2017 |  |  |  |  |  |  |  |  |  |  |  |  |  |  |  |  |  | |  |  |  |
| Tu, 2015 |  |  |  |  |  |  |  |  |  |  |  |  |  |  |  |  |  | |  |  |  |
| Tu, 2018 |  |  |  |  |  |  |  |  |  |  |  |  |  |  |  |  |  | |  |  |  |
| Wang, 2009 |  |  |  |  |  |  |  |  |  |  |  |  |  |  |  |  |  | |  |  |  |
| Wang, 2014 |  |  |  |  |  |  |  |  |  |  |  |  |  |  |  |  |  | |  |  |  |
| Wang, 2016 (a) |  |  |  |  |  |  |  |  |  |  |  |  |  |  |  |  |  | |  |  |  |
| Wang, 2016 PlosOne |  |  |  |  |  |  |  |  |  |  |  |  |  |  |  |  |  | |  |  |  |
| Wang, 2018 |  |  |  |  |  |  |  |  |  |  |  |  |  |  |  |  |  | |  |  |  |
| Wang, 2020 |  |  |  |  |  |  |  |  |  |  |  |  |  |  |  |  |  | |  |  |  |
| Wang, 2021 |  |  |  |  |  |  |  |  |  |  |  |  |  |  |  |  |  | |  |  |  |
| Xia, 2019 |  |  |  |  |  |  |  |  |  |  |  |  |  |  |  |  |  | |  |  |  |
| Xu, 2016 |  |  |  |  |  |  |  |  |  |  |  |  |  |  |  |  |  | |  |  |  |
| Xue, 2015 |  |  |  |  |  |  |  |  |  |  |  |  |  |  |  |  |  | |  |  |  |
| Yang, 2018 |  |  |  |  |  |  |  |  |  |  |  |  |  |  |  |  |  | |  |  |  |
| Yong-Hui, 2014 |  |  |  |  |  |  |  |  |  |  |  |  |  |  |  |  |  | |  |  |  |
| Yu, 2013 |  |  |  |  |  |  |  |  |  |  |  |  |  |  |  |  |  | |  |  |  |
| Yueh-Ling, 2012 |  |  |  |  |  |  |  |  |  |  |  |  |  |  |  |  |  | |  |  |  |
| Zhang, 2014 |  |  |  |  |  |  |  |  |  |  |  |  |  |  |  |  |  | |  |  |  |
| Zhang,2018 |  |  |  |  |  |  |  |  |  |  |  |  |  |  |  |  |  | |  |  |  |
| Zhao, 2020 |  |  |  |  |  |  |  |  |  |  |  |  |  |  |  |  |  | |  |  |  |
| Zhu, 2017 |  |  |  |  |  |  |  |  |  |  |  |  |  |  |  |  |  | |  |  |  |

Green: complete; Yellow: partial; Red: Incomplete; Abst: abstract; Bgrnd: background; Outc: outcomes
