# Supplementary material for: Effect of Physiotherapeutic Interventions on Biomarkers of Neuropathic Pain: A Systematic Review of Preclinical Literature
Source: J Pain. Author manuscript; Available in PMC 2022 Nov 3. (PMC7613788; doi:10.1016/j.jpain.2022.06.007)
Supplement: Supplementary Information [file EMS152413-supplement-Supplementary_Information.zip › 1-s2.0-S1526590022003509-mmc2.docx]

**Supplementary 2.** Characteristics of the included studies (I)

|  | Animals | NeuP Model | Groups | Intervention |
| --- | --- | --- | --- | --- |
| Chang, 2013 | Sprague-Dawley rats, male, 200-250g | Nerve crush injury; NC-sciatic nerve NeuP not confirmed by behavioural test | NC NC + acupuncture | **Acupuncture:** POD 24.   30 min with a slow rotation every 5 mi. GB30 Once a day for 5 days |
| Wang, 2009 | Sprague-Dawley, male, adult, 220 ± 10g | Chronic constriction injury; CCI-L5 NeuP confirmed by behavioural test | CCI CCI+ acupuncture | **Acupuncture:** POD 1 lumbago  pain, hip pain“ point. Daily for 30 min 14 days |
| Cha, 2010 | Sprague-Dawley rats, male, young adult, 200-250g | Nerve transection; NT-Sural and tibial nerves NeuP confirmed by behavioural test | NT NT+EA | **Electroacupuncture:** POD 14. 1 Hz, 0.1 ms pulse, 0.6 mA, 10 min, acupuncture points ST36 (Choksamni) and SP9 (Eumleungcheon). ?weeks ?sessions |
| Cha, 2012 | Sprague-Dawley rats, male, adult, 220-250g | Nerve transection; NT-sural and tibial nerves NeuP confirmed by behavioural test | NT NT+EA | **Electroacupuncture:** POD 14. 1 Hz, 0.1 ms pulse 0.6 mA, 10 min, acupuncture points ST36 (Choksamni) and SP9 (Eumleungcheon). ?weeks ?sessions |
| X.-M. Chen, 2015 | Sprague-Dawley rats, male, adult, 200-250g | Chronic constriction injury; CCI-Sciatic nerve NeuP confirmed by behavioural test | CCI CCI+EA | **Electroacupuncture**: POD 1, 2 Hz, 2 mA, 30 min, acupuncture points GB 30 (Huantiao) 2 weeks, ?sessions |
| Dong, 2005 (a) | Sprague-Dawley rats, male, adult, 200-220g | Chronic constriction injury CCI-Sciatic nerve NeuP not confirmed by behavioural test | CCI CCI+EA | **Electroacupunctur**e: POD 7, ≤1 mA, 60 Hz for 1.05 s and 2Hz for 2.85 s alternately, 30 min, acupoints GB-30 and GB-34 Daily to the end of experiment |
| Dong, 2005 (b) | Sprague-Dawley rats, male, adult, 200-220g | Chronic constriction injury CCI-Sciatic nerve NeuP confirmed by behavioural test | CCI CCI+EA | **Electroacupunctur**e: POD 7, ≤1 mA, 60 Hz for 1.05 s and 2Hz for 2.85 s alternately, 30 min, acupoints GB-30 and GB-34 Daily to the end of experiment |
| Liang, 2015 | Sprague-Dawley, male, 220–260 | Chronic constriction injury; CCI-L5 NeuP confirmed by behavioural test | CCI CCI+EA | **Electroacupuncture:** POD 1 . square wave current output (pulse width: 0.6 ms at 2 Hz, 0.2 ms at 100 Hz); intensities from 1 to 2 mA (15 min each, total 30 min); 2 and 100 Hz alternating frequencies y shifting between 2 and 100 Hz stimulation for 3 s each). ST36 bilaterally. BL60 bilaterally 11 days daily session. |
| Liang, 2016 | Sprague–Dawley rats, male, 220–240 g | Chronic constriction injury; CCI L4-L6 spinal nerves NeuP confirmed by behavioural test | CCI CCI + EA CCI + sham EA | **Electroacupuncture**: POD 1. square wave: 0.2 ms pulse intensities from 1to 2 mA (15 min each, totalling 30 min) at alternating (automatically shifting every 3 s) frequencies of 2and 100 Hz. Bilateral ST36 and BL60The stimulation was given at 24, 48 and72 h once per day. |
| Liu, 2019 | Sprague-Dawley rats, male, 180-220g | Chronic constriction injury; CCI-sciatic nerve NeuP confirmed by behavioural test | CCI CCI+EA | **Electroacupuncture:** POD 1,,” . 2 Hz/100Hz pulses for 30 minutes. The intensity ≤ 1 mA. “Weizhong Acupoint” and “Huantiao Acupoint 3 days 1 w |
| Shao, 2015 | Sprague-Dawley, male, adult 70 days old, 220–250 g | Chronic constriction injury; CCI- L5 NeuP confirmed by behavioural test | CCI EA strong manual acupuncture mild manual acupuncture | **Electroacupuncture**: POD 3 GB30. 0.6ms at 2Hz, 0.2ms at 100Hz,; intensities at 1 ± 0.5mA; alternating frequencies of 2Hz and 100Hz. 2min at a rate of 180 times per min, followed by an interval of 13min with needles retained and then another 2 min twisting stimulation. Daily session, 9 days. |
| Sun, 2004 | Sprague–Dawley, Male, 230–250g | Chronic constriction injury; CCI-L5 and L6 spinal nerves NeuP confirmed by behavioural test | CCI+ PES CCI+needling | **Peripheral electrical stimulation**, POD 8 frequency 2 Hz, pulse 0.6 ms. The intensity 3,increasing order (0.5, 1 and 2 mA) 10 min each, ST 36 , and Jiaji on both sides. ST 36 and Jiaji on the same side. 30 min. Once every 4 days 10 session |
| Tu, 2015 | Sprague Dawley, male, 200–250 g | Chronic constriction injury; CCI- sciatic nerve NeuP confirmed by behavioural test | CCI CCI + EA | **Electroacupuncture:** POD 7. square wave at 2/100Hz, intensity 2 mA for 30 min. ST36 and GB34. Daily session, 1 week. |
| Tu, 2018 | Sprague Dawley, male, 200-250g | Chronic constriction injury; CCI- sciatic nerve NeuP confirmed by behavioural test | CCI CCI+EA | **electroacupuncture:** POD 8. low (2 Hz) and high (100 Hz) frequencies 2/100 EA ST-36 and GB-34. Daily session, 1 week |
| Wang, 2014 | Sprague Dawley, male, 130–150g | Chronic constriction injury; CCI- sciatic nerve NeuP confirmed by behavioural test | CCI CCI + contralateral EA CCI +ipsilateral EA | **Electroacupuncture:** POD 7. 4 Square wave at 2/100 Hz, intensity 2 mA 30 min ST-3 and GB-3. Daily session, 1 week |
| Wang, 2016 | Wistar, male, adult, 200–250 g | Chronic constriction injury; CCI- sciatic nerve NeuP not confirmed by behavioural test | CCI CCI + sham EA CCI +EA | **Electroacupuncture:** POD 5. (2/100Hz, 1mA) was applied for 30 min ST36 and GB34 daily, for 2 weeks |
| Wang, 2018 | Sprague–Dawley, male, adult, 180–220 g | Chronic constriction injury; CCI-sural and tibial nerve NeuP confirmed by behavioural test | CCI CCI+EA | **Electroacupuncture: POD** 1. frequency 2 Hz pulse 0.6 Ms, intensity increased in a stepwise manner at 1–2–3 mA, each lasting for 10 min.30. min ST36 and SP6. Daily session, 3 weeks |
| Xia, 2019 | Sprague-Dawley, male, adult 7 to 8 weeks old, 160–180g | Chronic constriction injury; CCI-sural and tibial nerve NeuP not confirmed by behavioural test | CCI CCI+EA | **Electroacupuncture:** POD1 day. 2Hz pulse f 0.6 Ms, at 1-2-3 mA, each intensity 10 min ST36 Daily 21 days. |
| Xu, 2016 | Sprague-Dawley, male, adult, 200 to 250 g | Chronic constriction injury; CCI- sciatic nerve NeuP confirmed by behavioural test | CCI CCI+EA | **Electroacupuncture:** POD 1 A 2-Hz frequency and a 2-mA. 30 minutes. GB30. Daily session, 2week |
| Xue, 2015 | Sprague Dawley, male, 220-250g | Chronic constriction injury; CCI- L5 NeuP confirmed by behavioural test | CCI CCI+EA | **Electroacupuncture:**  POD 7. 2 Hz -1 mA 30 min. Huantiao and zusanli Daily session, 2week. |
| Yong-Hui, 2014 | Wistar, male, 240–300 g | Chronic constriction injury; CCI- sciatic nerve NeuP confirmed by behavioural test | CCI CCI +3 EA CCI+5EA CCI+12EA | **Electroacupuncture:** POD 7 1 mA and 15 Hz, 30 min ST36 and GB34. once daily continuously for 3, 5 or 12 consecutive days |
| Yu, 2013 | Sprague Dawley, male, 200 ± 20 | Chronic constriction injury; CCI- sciatic nerve NeuP confirmed by behavioural test | CCI group CCI+low-frequency EA CCI+ high-frequency EA | **Electroacupuncture:** POD 4. frequency 2 Hz or 15 Hz intensity f less than 1.5 mA. 30 min ST36 and GB34. Daily session, 5 days. |
| Zhang, 2014 | Sprague- Dawley, male, 220 ± 1.38g | Nerve transection; NT-C4/T1 NeuP confirmed by behavioural test | NT NT+EA | **Electroacupunctur**e: POD 2 . Dilatational wave (8 mA, 2–100 Hz) 15–20 minutes. LI11, LI04, ST36, GB34. Alternating the injured and uninjured sides on different days of the week. 3 sessions/week 2 weeks. |
| Zhang,2018 | Sprague-Dawley, male, 200–250, | Chronic constriction injury; CCI- sciatic nerve NeuP confirmed by behavioural test | CCI CCI+EA | **Electroacupuncture:** POD 7. 1 mm intensity and 2/15 Hz frequency11. 30 minutes. St 36and Lr 3. ? Session, ? week |
| Almeida, 2015 | BALB/c mice, male, 23.5-60.24g | Chronic constriction injury; CCI-Sciatic nerve NeuP confirmed by behavioural test | CCI CCI+ Swimming CCI+ Swimming+ Detraining | **Swimming:** POD7s. 12 in 3 sessions of 10, 20, 30, 40 minutes and 13 of 50 minutes). 25 session 5 ssesions/week 2 day rest |
| Bobinsky, 2011 | Swiss mice, male, 8-9 weeks old,25-35g | Nerve crush injury; NC-Sciatic nerve NeuP confirmed by behavioural test | Non-Exer NC+ Exercise-preoperative (Exer 1) NC+ Exercise-preoperative-postoperative (Exer 2) NC+ Exercise-postoperative (Exer 3) | **Low-intensity aerobic treadmill :**POD 3, 30 min at 10m/min with no inclination 5 days per week 2 weeks |
| Bobinsky, 2015 | Swiss mice, male, 8-9 weeks old,25-35g | Nerve crush injury; NC-Sciatic nerve NeuP confirmed by behavioural test | NC+ Sedentary NC+ Exercise | **Low-intensity aerobic treadmill:**, POD 3, k for 30 min at 10m/min with no inclination. 5 days per wee |
| Bobinsky, 2018 | Swiss mice, male, 20-30g | Nerve crush injury; NC-Sciatic nerve NeuP confirmed by behavioural test | NC+ Sedentary NC+ Exercise | **Low-intensity aerobic treadmill:**, POD 3, 5 for 30 min at 10m/min with no inclination days per week |
| Y-W. Chen, 2012 | Sprague-Dawley rats, male, 250-300g | Chronic constriction injury; CCI-Sciatic nerve NeuP confirmed by behavioural test | CCI CCI+ Swimming Exercise (CCISE) CCI+ Treadmill Exercise (CCITE) | **Swimming**: POD 1,, starting with 9 sessions of 10 min /15 min rest increasing day 7: 1 session of 90 min without res. 39 weeks, 5 days per week  **Treadmill:**  POD 1 , starting at 1.2 km/h during 15-30 min increasing 1.8 km/h during 60 min at days 33-39. 39 weeks, 5 days per week |
| Cobianchi, 2010. | CD1 mice, Male, 40-45 gr | Chronic constriction injury; CCI-sciatic nerve NeuP confirmed by behavioural test | CCI CCI+EX day3-7 CCI+Ex day3-56 | **TREADMILL**. POD 3 20cm/s that were increased 2 cm/s every 5 min (cut off 60 min). speed at the end of running was 52 cm/s. Running continued until exhaustion. 5 dasy/week 53 days. |
| Cobianchi, 2013* | Sprague-Dawley rats, female, adult, 210-270g | Nerve transection; NT-Sciatic nerve NeuP confirmed by behavioural test (only in Treadmill) | NT NT+TR | Electrical stimulation: start immediately, 1 session, 0.1 Ms, 3V, 2Hz, 4 h, anode: needle near muscle, cathode: wire bared at the tip **Treadmill** POD, 5 e days, 10 cm/s increasing 2 cm/s every 5 min until 32 cm/s, 1h |
| Coradini, 2015 | Wistar, male, 73 ± 4 days | Chronic constriction injury; CCI Median nerve NeuP confirmed by behavioural test | CCI  CCI+ Swim  CCI (Obese)  CCI+ Swim (Obese) | **Swimming**: POD 3  loads adjusted for swimming. first week, started with 20 min  second week 30 min and in the third week they exercised for 40 min, with a load of 10% of BW. Daily 5/week |
| Gong, 2017 | Wistar, male, 10 days | Chronic constriction injury; CCI-tibial and peroneal NeuP confirmed by behavioural test | CCI CCI+ exercise | **Treadmill:** Initiated 11 days after the injury. Postnatal day (P) 21P.  21-23 (P): 5m/min x 10 min 24-26 (P): 8m/min x 20 min 28-33 (P): 10m/min x 30 min 35-40 (P): 15m/min x 30 min |
| Huang, 2017 | Sprague-Dawley rats, male, 220-270g | Chronic constriction injury; CCI of the sciatic nerve NeuP confirmed by behavioural test | CCI CCI + TU0 CCI + TU CCI + TE CCI + TU0 + TE CCI + TU + TE | **Ultrasound:** POD 8 1 MHz with pulse (20% duty cycle) 1 W/cm2 intensity and 100-Hz frequency (beam no uniformity ratio = 3.6) for 5 minutes a day TU0: therapeutic ultrasound turned off **TE: Treadmill exercise**. 30 minutes Starting on postoperative day 8 and lasting daily for the next 3 weeks |
| Hung, 2014 | Sprague-Dawley rats, male, 220-270g | Chronic constriction injury; CCI-Sciatic nerve NeuP confirmed by behavioural test | CCI CCI+TT CCI+TU CCI+TT+TU | **Treadmill Training** :POD 3,, at 14-16 m/min, 8% inclination, 30 min 5 days per week during 4 weeks **Ultrasound :**POD 3,, 1 MHz with pulse (20% duty cycle), 1-W/cm2 intensity, 100-Hz frequency 5 minutes. 5 days per week during 4 weeks |
| Hung, 2016 | Sprague-Dawley rats, male, 220-270g | Chronic constriction injury; CCI-sciatic nerve NeuP confirmed by behavioural test | CCI CCI+TU CCI+TT CCI+TT+TU | **Ultrasound: T**he parameters were 1 MHz with pulse (20% duty cycle), 1-W/cm2 intensity, and 100-Hz frequency (beam no uniformity ratio 3.6) for 5 minutes a day. **TT: treadmill training: POD 3**t 14 to 16 m/min with an 8% incline for 30 minutes.  5 days a week for the next 4 weeks. |
| Kami, 2016a | Adult C57BL/6 J mice | CCI partial sciatic nerve ligation; CCI_P-sciatic NeuP confirmed by behavioural test | CCI-sedentary CCI + running | **Treadmill**: POD 2. 1º week 7 m/min for 10 min/day ,2º week 7 m/min for 20–60 min/day 3º week7 m/min for 60 min/day. 5 days/week |
| Kami, 2016b (Jpain) | Adult C57BL/6 J mice, male, 12 week | CCI partial sciatic nerve ligation; CCI_P-sciatic NeuP confirmed by behavioural test | CC_PI-sedentary CCI _P+ running | **Treadmill**: POD 2. 1º week 7 m/min for 10 min/day ,2º week 7 m/min for 20–60 min/day 3º week7 m/min for 60 min/day. 5 days/week |
| Korb 2010 | Wistar, adult male, 200-250g | Nerve transection; NT- Sciatic nerve NeuP confirmed by behavioural test | NT+ trained NT sedentary | **Treadmill:** r 20 min on the first day, increased every day up to 50 min 5 day and 60 min in the next 4 weeks. warm-up period of 5 min running at 30% of the maximal speed reached in the MET (5.5 m/min), 10–50 min running at 45–55% (*9 m/min) and 5 min recovery at 30% again (5.5 m/min), 5 sessions per week, once a day during 4 weeks. |
| López-Álvarez, 2015. | Sprague-Dawley, female, 240 +- 30 g | Chronic constriction injury; CCI- sciatic nerve NeuP confirmed by behavioural test | CCI+ITR1 CCI+ITR2 CCI | **Treadmill:** starting speed of 10 cm/s increased 2 cm/s every 5 minutes, until a maximal speed ofv32 cm/s. 60 min.. G1 POD3 daily session 5 days. G 2 POD 10-14 |
| López-Álvarez, 2018. | Sprague-Dawley rats, female, 240 ± 30 g | Sciatic nerve transection and repair SNTR NeuP confirmed by behavioural test | SNTR-iTR  SNTR-sedentary | **Treadmill:** POD 3startting speed of 10 cm/s increased 2 cm/s every 5 minutes, until a maximal speed ofv32 cm/s. 60 min. daily session 12 days |
| Martins, 2017. | Swiss mice, 8 weeks old, Male, 25-30 gr | Nerve crush injury; NC-sciatic NeuP confirmed by behavioural test | NC NC+ eccentric exercise 6 m/min  NC + eccentric exercise 10 m/min  NC + eccentric exercise 14 m/min | **Eccentric:** (Downhill Running) Program:30 min at a speed of 6, 10, or 14 m/min with − 16° slope, 5 days per week, for 8 weeks. |
| Sumizono, 2018 | Sprague Dawley, male, 8 weeks, 274.3 ± 21.2 g | Chronic constriction injury; CCI-sciatic nerve NeuP confirmed by behavioural test | CCI CCI + high-frequency exercise CCI + low-frequency exercise | **Treadmill**: speed of 20 m/min, for either 5 days (HFE) or 3 days (LFE) a week, for a total of 5 weeks |
| Tian, 2018 | Sprague-Dawley rats, male, 200-250g | Nerve transacted; NT- Tibial nerve NeuP confirmed by behavioural test | NT NT+ swimming | **Swim**: POD 7 1ºweek,10-min swimming exercise 1º day, gradually increased r 60 min. In the following 4 wk, rats swam daily for 5 d followed by 2-d rest.  weeks swimming exercise |
| Tsai, 2017 | Sprague-Dawley, male, 285–335 g. | Chronic constriction injury; CCI- sciatic nerve NeuP confirmed by behavioural test | CCI CCI + 0%-incline treadmill CCI + 8%-incline treadmill | **Treadmill:**  POD 6. 14–16 m/min with/without 8% incline grade for 30 min . Daily sessions 3 weeks |
| Wang, 2016 | New Zealand white rabbit, male, 11±12 weeks-old, 1.78 ±0.12 kg | Nerve crush injury; NC-sciatic nerve NeuP not confirmed by behavioural test | NC NC+Ex NC+EX+EA | **Treadmill:** POD 3 for 20 minutes a day at a rate of 10m/min for 3 days, at a rate of 15m/min for 20 min/day from the fourth to sixth day, and then at a rate of 20m/min for 20 min/day from the seventh day onward, 6 days/week for a total treatment cycle duration of 4 weeks  **EX+EA:**3 days post-surgery, 6 Jiaji acupoints3Hz/s electrical current for 30 min/day, 6 days/week for a total treatment duration of 4 weeks |
| Martins, 2011 | Wistar rats, male, adult, 250-280g | Nerve crush; NC-Sciatic nerve NeuP confirmed by behavioural test | NC NC+Anesthesia NC+AJM | **Ankle joint mobilisation:** POD1 dorsal and plantar flexion,, s, 3 treatments of 3 minutes with 30 seconds rest, 48 h rest between sessions. 15 session |
| Song, 2016 | Sprague-Dawley, male, adult, 200-250 | Chronic constriction injury and decompresion CCI-DRG de-CCI-DRG NeuP confirmed by behavioural test | CCI de-CCI de-CCI+SMT | **Chiropractic spinal manipulative therapy**. POD10. b0.1 ms mechanical force, manually assisted spinal manipulative thrusts, L5 and L6. 10 ASMT. Daily for consecutive 5 days |
| da Silva, 2015 | Wistar rats, male, two weeks, 180-220g | Chronic constriction injury; CCI-Sciatic nerve NeuP not confirmed by behavioural test | CCI CCI+NM | **Neural mobilisation:** POD 14, 10 sessions, 20 oscillations per minute during 2min, followed by a 25-s pause, total of 10 minutes, ankle joint in dorsiflexion (30–45 degrees) |
| Giardini, 2017 | Wistar, male, 2 month, 200-220g | Chronic constriction injury; CCI-sciatic nerve NeuP not confirmed by behavioural test | CCI CCI+NM | **Neural Mobilization:** POD14 20 oscillations per minute for 2 minutes 25-second rest. ten minutes total. initiated 14 days after the injury. Every other day for a total of 10 sessions. |
| Santos, 2012 | wistar, male, 2 months-old,180 and 220 g | Chronic constriction injury; CCI-sciatic nerve NeuP confirmed by behavioural test | CCI CCI+ NM | **Neural Mobilization**: POD14. 20 oscillations/ min 2 min, 25-sr rest.10 min total last minute the cervical spine was fully flexed, 10 sessions. |
| Santos, 2018 | wistar, male, 200 and 220 g | Chronic constriction injury; CCI-sciatic nerve NeuP not confirmed by behavioural test | CCI CCI+ NM | **Neural Mobilization**: 10 sessions. 20 oscillations/ min 2 min, 25-sr rest. |
| Zhu, 2017 | Sprague-Dawley, male, mature, 300 to 320g | Diabetes Neuropthy NeuP confirmed by behavioural test | Diabetes Diabetes +neural mobilization | **Neural Mobilization:** POD 10. . 20 times/min. 2-min oscillation, 25-s break; 5 repetitions for each treatment session. 1 session/d, 5 d/wk. 3 weeks |
| Chen, 2015 | Sprague–Dawley, male, 200–250 g | Chronic constriction injury; CCI- sciatic nerve NeuP confirmed by behavioural test | CC I CCI+ TU-0 CCI + TU-0.25 CCI+TU-0.5 CCI+TU-1 | **Ultrasound:** POD **5.** 1-MHz frequency intensity of 70.25, 0.5 or 1 W/cm2 and 100% on–off cycle 5 min once a day, 22 days |
| Cidral, 2013 | Swiss mice, male, adult, 25-35g | Nerve crush injury; NC-Sciatic nerve NeuP confirmed by behavioural test | NC NC+LEDT | **Light-emitting diode therapy:** POD 7, 950 nm, 80 mW/cm2 and 2.5 J/cm2, Daily sessions 15 days. |
| Cioato, 2016 | Wistar rats, male, 55-65 days, 200-250g | Chronic constriction injury; CCI-Sciatic nerve NeuP confirmed by behavioural test | CCI CCI+s ham tDCS CCI+ tDCS | **Transcranial direct current stimulation:** POD15. , 0.5 mA, 1.5 cm2 electrodes, 20 min, anode: head, cathode: supraorbital area. Daily 8 day |
| Filho, 2016 | Wistar rats, male, 55-65 days, ≥ 250g | Chronic constriction injury; CCI-Sciatic nerve NeuP not confirmed by behavioural test | CCI CCI+ Sham tDCS CCI+t DCS | **Transcranial direct current stimulation:**,POD 14, 8 days, 0.5 mA, 1.5 cm2 electrodes, 20 min, anode: parietal cortex, cathode: supraorbital area. Daily 8 days |
| Giuliani, 2004 | Sprague-Dawley rats, male, 2 month, 250-275g | Chronic constriction injury; CCI-sciatic nerve NeuP confirmed by behavioural test | CCI CCI + laser | **Laser:** POD 8 power 0.03 mW, wavelength of 670nm, 1% duty cycle modulation of a 3-mW peak power diode, frequency 100Hz.  each point lasted 35 sec (1.05mJ) t area is less than 5mm2. Density less than 0,21 mJ/mm2  2 selected points every 3 days |
| Hsieh, 2012 | Sprague-Dawley rats, male, 250-300g | Chronic constriction injury; CCI-sciatic nerve NeuP confirmed by behavioural test | CCI+laser CCI+sham | **Laser:** POD 7  continuous 660 nm Ga-Al-  or sham size was 0.2 cm2 aprox. power irradiation was 30 mW per session for 60 seconds per spot, 4 spots . The energy density was 9 J/cm2. Control group with power set at 0. daily 1 week |
| Lin, 2015 | Sprague-Dawley rats, male, 200-250g | Chronic constriction injury; CCI- sciatic nerve NeuP confirmed by behavioural test | CCI CCI+ HFS | **TENS**: POD 1 80% of that enough to elicit an obvious muscle contraction. The pulse duration was set at 100us for 20 min Daily 13 d |
| Liu, 2017 | Sprague-Dawley rats, male, 300-350g | Chronic constriction injury; CCI- sciatic nerve NeuP confirmed by behavioural test | CCI + sham PEMF CCI + PEMF | **Pulsed electromagnetic field therapy:** POD 1 Set as 3.8mT, 8Hz, 30min. The treatment began from the first day and sustained 2 weeks |
| Matsuo, 2014. | ICR mice (Clea, Tokyo), 9 weeks, male, 39.6±3.0 g | Chronic constriction injury; CCI-tibial and peroneal nerves NeuP confirmed by behavioural test | CCI CCI+TENS 1 w CCI+TENS 2 w | **TENS:** POD 7   frequency 100 Hz and the intensity defined by the sensory threshold. 30min. Of 1w or 2w |
| Mert, 2015 (a). | Wistar rats, 8-12 weeks, Male, 270-280 gr | Chronic constriction injury; CCI-sciatic nerve NeuP confirmed by behavioural test | sham PMF (SPMF)   PMF-AD PMF-AW | **Pulse magnetic field:** POD 1- 7. Three sequences. Each sequence included four different consecutive pulse trains (1, 10, 20, 40 Hz). The each pulse train was 4 min, and the interval 1 min. 60 min. dailyl for 4 weeks |
| Mert, 2017 | Wistar rats, 10-12 weeks, Male, 280-290 gr | Chronic constriction injury; CCI-sciatic nerve NeuP confirmed by behavioural test | CCI+PMF  CCI+SPMF | **Pulsed Magnetic Field:** POD1 Three sequences. Each sequence included four different consecutive pulse trains (1, 10, 20, 40 Hz). The each pulse train was 4 min, and the interval 1 min. 60 min. daily for 4 weeks. |
| Somers, 2003 | Sprague-Dawley rats, male, adult, 150-165g | Chronic constriction injury; CCI-Sciatic nerve NeuP not confirmed by behavioural test | CCI CCI+TENS | **High-Frequency TENS,** POD 0 , s, 30 to 40 uA, 100Hz, 60 min per day, Para spinal musculature. 11 days. |
| Somers, 2009 | Sprague-Dawley rats, male, 150 to 175 g | Chronic constriction injury; CCI-sciatic nerve NeuP not confirmed by behavioural test | CCI CCI+ high frequency TENS contralateral CCI + low-frequency TENS CCI +randomly TENS | **Electrical Nerve Stimulation**: POD 0 s. 1 day90 min 10 days 60 min 100 Hz high frequency 30-40 microamperes delivered through 45 mm 5-mm electrodes 2 Hz Low frequency 30-40 microamperes delivered through 45 mm 5-mm electrodes 11 Daily days |
| Su, 2018 | Sprague-Dawley rats, male, 250-300g | Nerve crush injury; NC- sciatic nerve NeuP confirmed by behavioural test | NC NC + High-frequency immediately(HFI) NC + High-frequency 7 days after(HFL) NC + Low-frequency immediately (LFI) NC + Low-frequency 7 days after (HFL) | **Electrical stimulation:** POD 400 ms of biphasic pulses at 200 μs per phase. HF use 100hz and LF 5 Hz frequency and with 6 s of rest 30 min per day for 1ws |
| Yang, 2018 | Sprague-Dawley, male, 180 to250 g | Chronic constriction injury; CCI- sciatic nerve NeuP confirmed by behavioural test | CCI+ sham-rTMS group CCI+ 1 Hz group CCI+ 20 Hz group | **Transcranial Magnetic Stimulation:** POD 3. right primary motor cortex (M1) contralateral to the pain side with 90% RMT stimulation intensity (40% of the maximum output), and 1600 pulses for each treatment. 1 Hz group was 1 Hz with continuous stimulation for total 26.7 min, and that of 20 Hz group was 20 Hz, with 4 s for each sequence and a 30 s interval, 20 sequences in total for each treatmen Daily 10 days |
| Yueh-Ling, 2012 | Sprague–Dawley, male, adult, 250–300 g | Chronic constriction injury; CCI- sciatic nerve NeuP confirmed by behavioural test | CCI and treated with laser CCI and treated with sham irradiation | **Laser:** POD 7 day. continuous 660 nm Ga-Al-As diode laser. Size 0.2 cm2. 30 mW per session for 60 seconds per spot . The energy density was 9 J/cm 7 days |

| Wang, 2020 | Sprague–Dawley, rats, male  160–180 g, 7-8 weeks | Spared nerve injury  NeuP confirmed by behavioural test | Sham  Injury+EA  Injury | POD 3, 2Hz EA, 1-2-3mA, once a day for 30 min, every other day. for 3 weeks. |
| --- | --- | --- | --- | --- |
| Li, 2019 | Sprague–Dawley rats, male, 5–8 weeks, 180–220 g | Model of paclitaxel-induced peripheral neuropathic pain CIPN Antineoplastic neuropathy NeuP confirmed by behavioural test | CIPN CIPN + EA CIPN + sham EA | **Electroacupuncture**: 2 Hz, square wave  0.2 ms pulse from 0.5 to 1.5mA (increased by 0.5 mA every 10 min, for a total of 30 min). Needles of 0.25mm, inserted 5 mm in bilateral ST36 and BL60.Once daily for 7 consecutive days. |
| Hsieh, 2017 | Sprague-Dawley rats, male, 200-250g | Oxaliplatin administration: Antineoplasic neuropathy NeuP not confirmed by behavioural test | Oxaliplatin +TUS Oxaliplatin +shamTUS | **Ultrasound**: POD 4 hours Pulsed-mode TUS (1 MHz, spatial average/temporal average intensity [ISATA] 5 0.5 W/cm2, 50% duty cycle) was applied for 5 min. sonication/non-sonication times of 2 ms/2 ms.  Daily 10 sessions. |
| Zhao, 2020 | Sprague– Dawley, rats, male 120– 150 g | Paclitaxel (PTX)  NeuP confirmed by behavioural test | Control group  PTX group  PTX +  EA group  PTX + sham EA group | POD 3 Acupoints of ST36 (Zusanli) on bilateral hind limbs  10 mm below the knee joint and 5 mm lateral to  the anterior tubercle of the tibia. 10 Hz, 1 mA) were applied for 30 min  EA procedures were performed under isoflurane  anesthesia for 14 days. |
| Belmonte, 2018 | Swiss mice, male, 40-50g | Complex pain syndrome; CPIP NeuP confirmed by behavioural test | CPIP CPIP+ Exercise continuous CPIP+ Exercise interval protocol | **Continous and interval treadmill :**, day 7, for 1, 3 alternate days or 5 consecutive days with 2 (two) days interval. Continuous exercise was at 16m/min. The interval exercise was 3:1 ratio at 16 and 19 m/min. Each session 30 min |
| Manni, 2011. | Sprague–Dawley rats, female, 200–220 g | Diabetes Neuropathy NeuP confirmed by behavioural test | 12 STZ group 12 STZ group +EA | **Electroacupuncture** POD 7 square wave with duration 0.18 ms, a burst length 0.1 s, and internal burst frequency of 80 Hz. The intensity (1.0–1.5 mA burst frequency of 2 Hz30 min bilaterally at the traditional acupoint Zusanl twice a week for 3 weeks. |
| Nori, 2013. | Sprague-Dawley rats, Female, 200-220 gr | Diabetes Neuropathy NeuP not confirmed by behavioural test | DN DN+EA | **Electroacupuncture:** POD 7 30 minutes  low burst frequency of 2Hz; pulse square electric wave f 180 𝜇sec, a length of 0.1 sec, and internal burst frequency of 80 Hz, intensity (1.0–1.5mA)  bilaterally ST36. twice a week for 3 weeks |
| Shi, 2013 | Sprague-Dawley, female, 160–180 g | Diabetes Neuropathy NeuP confirmed by behavioural test | Diabetes Diabetes +EA | **Electroacupuncture**: POD 21. 100 Hz for 1.05 s and 2 Hz for 2.85 s alternately, pulse 0.1 ms.30 min each day ST-361 week. |
| Y-W. Chen, 2013 | Wistar rats, male, 285-335g | Diabetes Neuropathy NeuP confirmed by behavioural test | Sedentary +DN Exercise +DN | **Treadmill:**, POD? starting at 20 m/min for 30 min and gradually increasing to 20 m/ min for 60 min first 2 weeks. ? Sessions/week 8 weeks |
| Y-W. Chen, 2015 | Wistar rats, male, 290-340g | Diabetes Neuropathy NeuP confirmed by behavioural test | Sedentary +DN Exercise +DN | **Treadmill:** POD3, , 1.2 km/h for 30 min (first 2 weeks), 1.5 km/h for 60 min (second 2 weeks) ? Sessions/week 4 weeks |
| Ma, 2018. | Sprague-Dawley rats, Male, 200-250 gr | Diabetes Neuroapathy NeuP confirmed by behavioural test | DN DN+EX | **Treadmill:** POD 4 . speed was gradually increased from 5 m/min at a 10% grade, and exercise duration was maintained at 10 min. By the third week, the intensity increased to 10 m/min for 10 min, 4 days/week for 5 weeks |
| Thakur, 2016 | SpragueDawley, male, 250–280 g | Diabetes Neuropathy NeuP not confirmed by behavioural test | 1diabetes 2diabetic+exercise | **Treadmill**: POD 14, speed10m/min, 60min per day five days/week, for 6 weeks break for waterafter20min |
| Mert, 2015 (b). | Wistar rats, 10-12 weeks, Male, 280-300 gr | Diabetes Neuropathy NeuP confirmed by behavioural test | STZ-induced diabetic L-PMF-treated diabetic H-PMF-treated diabetic | **Pulsed magnetic field**. POD3 Three sequences. Each sequence included four different consecutive pulse trains (1, 10, 20, 40 Hz). The each pulse train was 4 min, and the interval 1 min. 60 min. daily for 5 weeks |
| da Silva Oliveira, 2018 | C57BL6 mice, male, 20-26g | Diabetes Neuropathy NeuP confirmed by behavioural test | DN+ Sham DN+PBM | **Photobiomodulation:** POD 14. , 660 nm, 0.28 cm2, 30 mW, 1.6 J/cm2, 15 sec in a continuous frequency, plantar region of the left hind paw. 21 sessions |
| Tang, 2020 | Sprague–Dawley, rats, male  180–220 g, 7 weeks | Diabetic peripheral neuropathy ( streptozotocin)  NeuP confirmed by behavioural test | Control  Diabetic neuropathy  Diabetic neuropathy+acupuncture | POD ? Acupuncture points,  Feishu, Pishu and Shenshu. 20 min once daily for 14 days |
| Wang 2021, | Sprague–Dawley, rats, male  200-220 g, 8 weeks | Diabetic peripheral neuropathy ( streptozotocin)  NeuP confirmed by behavioural test | Control  Model  EA | POD ? continuous-wave stimulation for 20 min, with an alternating frequency of 2/15 Hz and a current of 2 mA. acupuncture needles (0.18 mm in diameter, 10 mm in length) were inserted at a depth of 3 mm. six times a week and lasted for 5  weeks. |

NC nerve crush; CCI chronic constriction injury ; NT nerve transection; CPIP chronic post-ischemia pain; STZ streptozocin DN diabetic neuropathy; SNTR sciatic nerve transection and repair; POD post operative day; ? not reported; PMF pulse magnetic field; SPMF sham pulse magnetic field; EX exercise; EA electro-acupuncture; AJM ankle joint mobilization; SMT spinal manipulative therapy; HFI High-frequency immediately; HFL Low-frequency immediately
