# Supplementary material for: Effect of Physiotherapeutic Interventions on Biomarkers of Neuropathic Pain: A Systematic Review of Preclinical Literature
Source: J Pain. Author manuscript; Available in PMC 2022 Nov 3. (PMC7613788; doi:10.1016/j.jpain.2022.06.007)
Supplement: Supplementary Information [file EMS152413-supplement-Supplementary_Information.zip › 1-s2.0-S1526590022003509-mmc3.docx]

| **Appendix 1****:** Database formulas during literature search |
| --- |
|  |
| **CINAHL Search Formula vía EBSCO.**  ("Models, Animal”[Mesh] OR “animal*” OR “mice” OR “rat*” OR “transgenic mice”) AND (“nerve block method*” OR “nerve crush” OR “nerve constriction” OR “nerve crush” OR “nerve cut” OR “nerve constriction” OR “nerve injury” OR “nerve ligation” OR “constriction pathologic*” OR “chronic constriction injury” OR “peripheral neuropathy” OR “nerve inflammation” OR “neuropathy” OR "diabetic*" OR "chemotherapy" OR "metabolic*") AND (“neuropathic pain” OR “Neuralgia” [Mesh] OR “neuropathic*” OR “sciatica” OR “pain”) AND (“Physical therapy modalities”[Mesh] OR “physical therap*” OR “physiotherap*” OR "Musculoskeletal manipulations" OR "Manual therap*” OR “exercise therapy” OR “exercise*” OR “run*” OR “swim*” OR “strength*” OR “endurance” OR “resistance” OR “physical conditioning” OR "neural tension technique*" OR “neural mobilization*” OR “neural mobilisation” OR “neural stretching” OR “neural gliding” OR “massage*” OR “massotherapy” OR “peripheral nerve therapy” OR "Electric Stimulation Therapy"[Mesh] OR electrotherap* OR “TENS” OR “Acupuncture”[Mesh] OR “dry needling” OR "manipulation*” OR ”mobilization*" OR "mobilisation*") AND (“biomarker*” OR “biological factor*” OR “nerve tissue protein” OR “nerve regeneration” OR “pain measurement” OR “glial cell line-derived neurotrophic factor” OR “glial cell line-derived neurotrophic factor receptor*” OR “brain-derived neurotrophic factor” OR “nerve growth factor receptor*” OR “receptor trkB” OR “nerve growth factor*” OR “neurotrophin*” OR “receptor trkA” OR “receptor trkC” OR “neuropeptide*” OR “cytokine*” OR “interleukin*” OR “interleukin receptor” OR “macrophage*” OR “immunology*” OR “glial cell*” OR “astrocyte*” OR “schwann cell*” OR “microglia” OR “oligodendroglia*” OR “satellite cell*”)  **PsycINFO Search Formula vía EBSCO:**  ("Models, Animal”[Mesh] OR “animal*” OR “mice” OR “rat*” OR “transgenic mice”) AND (“nerve block method*” OR “nerve crush” OR “nerve constriction” OR “nerve crush” OR “nerve cut” OR “nerve constriction” OR “nerve injury” OR “nerve ligation” OR “constriction pathologic*” OR “chronic constriction injury” OR “peripheral neuropathy” OR “nerve inflammation” OR “neuropathy” OR "diabetic*" OR "chemotherapy" OR "metabolic*") AND (“neuropathic pain” OR “Neuralgia” [Mesh] OR “neuropathic*” OR “sciatica” OR “pain”) AND (“Physical therapy modalities”[Mesh] OR “physical therap*” OR “physiotherap*” OR "Musculoskeletal manipulations" OR "Manual therap*” OR “exercise therapy” OR “exercise*” OR “run*” OR “swim*” OR “strength*” OR “endurance” OR “resistance” OR “physical conditioning” OR "neural tension technique*" OR “neural mobilization*” OR “neural mobilisation” OR “neural stretching” OR “neural gliding” OR “massage*” OR “massotherapy” OR “peripheral nerve therapy” OR "Electric Stimulation Therapy"[Mesh] OR electrotherap* OR “TENS” OR “Acupuncture”[Mesh] OR “dry needling” OR "manipulation*” OR ”mobilization*" OR "mobilisation*") AND (“biomarker*” OR “biological factor*” OR “nerve tissue protein” OR “nerve regeneration” OR “pain measurement” OR “glial cell line-derived neurotrophic factor” OR “glial cell line-derived neurotrophic factor receptor*” OR “brain-derived neurotrophic factor” OR “nerve growth factor receptor*” OR “receptor trkB” OR “nerve growth factor*” OR “neurotrophin*” OR “receptor trkA” OR “receptor trkC” OR “neuropeptide*” OR “cytokine*” OR “interleukin*” OR “interleukin receptor” OR “macrophage*” OR “immunology*” OR “glial cell*” OR “astrocyte*” OR “schwann cell*” OR “microglia” OR “oligodendroglia*” OR “satellite cell*”)  **Medline Search Formula (EBSCO).**  ("Models, Animal”[Mesh] OR “animal*” OR “mice” OR “rat*” OR “transgenic mice”) AND (“nerve block method*” OR “nerve crush” OR “nerve constriction” OR “nerve crush” OR “nerve cut” OR “nerve constriction” OR “nerve injury” OR “nerve ligation” OR “constriction pathologic*” OR “chronic constriction injury” OR “peripheral neuropathy” OR “nerve inflammation” OR “neuropathy” OR "diabetic*" OR "chemotherapy" OR "metabolic*") AND (“neuropathic pain” OR “Neuralgia” [Mesh] OR “neuropathic*” OR “sciatica” OR “pain”) AND (“Physical therapy modalities”[Mesh] OR “physical therap*” OR “physiotherap*” OR "Musculoskeletal manipulations" OR "Manual therap*” OR “exercise therapy” OR “exercise*” OR “run*” OR “swim*” OR “strength*” OR “endurance” OR “resistance” OR “physical conditioning” OR "neural tension technique*" OR “neural mobilization*” OR “neural mobilisation” OR “neural stretching” OR “neural gliding” OR “massage*” OR “massotherapy” OR “peripheral nerve therapy” OR "Electric Stimulation Therapy"[Mesh] OR electrotherap* OR “TENS” OR “Acupuncture”[Mesh] OR “dry needling” OR "manipulation*” OR ”mobilization*" OR "mobilisation*") AND (“biomarker*” OR “biological factor*” OR “nerve tissue protein” OR “nerve regeneration” OR “pain measurement” OR “glial cell line-derived neurotrophic factor” OR “glial cell line-derived neurotrophic factor receptor*” OR “brain-derived neurotrophic factor” OR “nerve growth factor receptor*” OR “receptor trkB” OR “nerve growth factor*” OR “neurotrophin*” OR “receptor trkA” OR “receptor trkC” OR “neuropeptide*” OR “cytokine*” OR “interleukin*” OR “interleukin receptor” OR “macrophage*” OR “immunology*” OR “glial cell*” OR “astrocyte*” OR “schwann cell*” OR “microglia” OR “oligodendroglia*” OR “satellite cell*”) |
| **PubMed Search Formula vía NLM.**  ("Models, Animal”[Mesh] OR “animal*” OR “mice” OR “rat*” OR “transgenic mice”) AND (“nerve block method*” OR “nerve crush” OR “nerve constriction” OR “nerve crush” OR “nerve cut” OR “nerve constriction” OR “nerve injury” OR “nerve ligation” OR “constriction pathologic*” OR “chronic constriction injury” OR “peripheral neuropathy” OR “nerve inflammation” OR “neuropathy” OR "diabetic*" OR "chemotherapy" OR "metabolic*") AND (“neuropathic pain” OR “Neuralgia” [Mesh] OR “neuropathic*” OR “sciatica” OR “pain”) AND (“Physical therapy modalities”[Mesh] OR “physical therap*” OR “physiotherap*” OR "Musculoskeletal manipulations" OR "Manual therap*” OR “exercise therapy” OR “exercise*” OR “run*” OR “swim*” OR “strength*” OR “endurance” OR “resistance” OR “physical conditioning” OR "neural tension technique*" OR “neural mobilization*” OR “neural mobilisation” OR “neural stretching” OR “neural gliding” OR “massage*” OR “massotherapy” OR “peripheral nerve therapy” OR "Electric Stimulation Therapy"[Mesh] OR electrotherap* OR “TENS” OR “Acupuncture”[Mesh] OR “dry needling” OR "manipulation*” OR ”mobilization*" OR "mobilisation*") AND (“biomarker*” OR “biological factor*” OR “nerve tissue protein” OR “nerve regeneration” OR “pain measurement” OR “glial cell line-derived neurotrophic factor” OR “glial cell line-derived neurotrophic factor receptor*” OR “brain-derived neurotrophic factor” OR “nerve growth factor receptor*” OR “receptor trkB” OR “nerve growth factor*” OR “neurotrophin*” OR “receptor trkA” OR “receptor trkC” OR “neuropeptide*” OR “cytokine*” OR “interleukin*” OR “interleukin receptor” OR “macrophage*” OR “immunology*” OR “glial cell*” OR “astrocyte*” OR “schwann cell*” OR “microglia” OR “oligodendroglia*” OR “satellite cell*”)  **Scopus Search Formula vía ELSEVIER:**   TITLE-ABS-KEY ( "animal*" OR "mice" OR "rat*" OR "transgenic mice" ) AND TITLE-ABS-KEY ( "nerve block method*" OR "nerve crush" OR "nerve constriction" OR "nerve crush" OR "nerve cut" OR "nerve constriction" OR "nerve injury" OR "nerve ligation" OR "constriction pathologic*" OR "chronic constriction injury" OR "peripheral neuropathy" OR "nerve inflammation" OR "neuropathy" OR "diabetic*" OR "chemotherapy" OR "metabolic*" ) TITLE-ABS-KEY ( "neuropathic pain" OR "Neuralgia" OR "neuropathic*" OR "sciatica" OR "pain" ) AND TITLE-ABS-KEY ( "Physical therapy modalities" OR "physical therap*" OR "physiotherap*" OR "Musculoskeletal manipulations" OR "Manual therap*" OR "exercise therapy" OR "exercise*" OR "run*" OR "swim*" OR "strength*" OR "endurance" OR "resistance" OR "physical conditioning" OR "neural tension technique*" OR "neural mobilization*" OR "neural mobilisation" OR "neural stretching" OR "neural gliding" OR "massage*" OR "massotherapy" OR "peripheral nerve therapy" OR "Electric Stimulation Therapy" OR electrotherap* OR "TENS" OR "Acupuncture" OR "dry needling" OR "manipulation*" OR "mobilization*" OR "mobilisation*" ) TITLE-ABS-KEY ( "biomarker*" OR "biological factor*" OR "nerve tissue protein" OR "nerve regeneration" OR "pain measurement" OR "glial cell line-derived neurotrophic factor" OR "glial cell line-derived neurotrophic factor receptor*" OR "brain-derived neurotrophic factor" OR "nerve growth factor receptor*" OR "receptor trkB" OR "nerve growth factor*" OR "neurotrophin*" OR "receptor trkA" OR "receptor trkC" OR "neuropeptide*" OR "cytokine*" OR "interleukin*" OR "interleukin receptor" OR "macrophage*" OR "immunology*" OR "glial cell*" OR "astrocyte*" OR "schwann cell*" OR "microglia" OR "oligodendroglia*" OR "satellite cell*" ) Wos Searh Formula vía ELSEVIER:  *Índices=SCI-EXPANDED, SSCI, A&HCI, CPCI-S, CPCI-SSH, BKCI-S, BKCI-SSH, ESCI, CCR-EXPANDED, IC Período de tiempo=Todos los años*  # 1 [**11.064.724**](https://apps.webofknowledge.com/summary.do?product=WOS&doc=1&qid=2&SID=D5hmJRulTs4crjeQmvg&search_mode=AdvancedSearch&update_back2search_link_param=yes) TS= ("animal*" OR "mice" OR "rat*" OR "transgenic mice")  *Índices=SCI-EXPANDED, SSCI, A&HCI, CPCI-S, CPCI-SSH, BKCI-S, BKCI-SSH, ESCI, CCR-EXPANDED, IC Período de tiempo=Todos los años*  # 2 [**1.380.208**](https://apps.webofknowledge.com/summary.do?product=WOS&doc=1&qid=3&SID=D5hmJRulTs4crjeQmvg&search_mode=AdvancedSearch&update_back2search_link_param=yes) TS=("nerve block method*" OR "nerve crush" OR "nerve constriction" OR "nerve crush" OR "nerve cut" OR "nerve constriction" OR "nerve injury" OR "nerve ligation" OR "constriction pathologic*" OR "chronic constriction injury" OR "peripheral neuropathy" OR "nerve inflammation" OR "neuropathy" OR "diabetic*" OR "chemotherapy" OR "metabolic*")  *Índices=SCI-EXPANDED, SSCI, A&HCI, CPCI-S, CPCI-SSH, BKCI-S, BKCI-SSH, ESCI, CCR-EXPANDED, IC Período de tiempo=Todos los años*  # 3 [**642.424**](https://apps.webofknowledge.com/summary.do?product=WOS&doc=1&qid=23&SID=D5hmJRulTs4crjeQmvg&search_mode=AdvancedSearch&update_back2search_link_param=yes) TS=(“neuropathic pain” OR"neuralgia" OR “neuropathic*” OR “sciatica” OR “pain”)  *Índices=SCI-EXPANDED, SSCI, A&HCI, CPCI-S, CPCI-SSH, BKCI-S, BKCI-SSH, ESCI, CCR-EXPANDED, IC Período de tiempo=Todos los años*  # 4 [**4.273.448**](https://apps.webofknowledge.com/summary.do?product=WOS&doc=1&qid=25&SID=D5hmJRulTs4crjeQmvg&search_mode=AdvancedSearch&update_back2search_link_param=yes) TS= (“Physical therapy modalities” OR “physical therap*” OR “physiotherap*” OR "Musculoskeletal manipulations" OR "Manual therap*” OR “exercise therapy” OR “exercise*” OR “run*” OR “swim*” OR “strength*” OR “endurance” OR “resistance” OR “physical conditioning” OR "neural tension technique*" OR “neural mobilization*” OR “neural mobilisation” OR “neural stretching” OR “neural gliding” OR “massage*” OR “massotherapy” OR “peripheral nerve therapy” OR "Electric Stimulation Therapy" OR electrotherap* OR “TENS” OR “Acupuncture” OR “dry needling” OR "manipulation*” OR ”mobilization*" OR "mobilisation*")  # 5 [**1.387.467**](https://apps.webofknowledge.com/summary.do?product=WOS&doc=1&qid=17&SID=D5hmJRulTs4crjeQmvg&search_mode=AdvancedSearch&update_back2search_link_param=yes) TS=(“biomarker*” OR “biological factor*” OR “nerve tissue protein” OR “nerve regeneration” OR “pain measurement” OR “glial cell line-derived neurotrophic factor” OR “glial cell line-derived neurotrophic factor receptor*” OR “brain-derived neurotrophic factor” OR “nerve growth factor receptor*” OR “receptor trkB” OR “nerve growth factor*” OR “neurotrophin*” OR “receptor trkA” OR “receptor trkC” OR “neuropeptide*” OR “cytokine*” OR “interleukin*” OR “interleukin receptor” OR “macrophage*” OR “immunology*” OR “glial cell*” OR “astrocyte*” OR “schwann cell*” OR “microglia” OR “oligodendroglia*” OR “satellite cell*”)  *Índices=SCI-EXPANDED, SSCI, A&HCI, CPCI-S, CPCI-SSH, BKCI-S, BKCI-SSH, ESCI, CCR-EXPANDED, IC Período de tiempo=Todos los años*  # 6 [**327**](https://apps.webofknowledge.com/summary.do?product=WOS&doc=1&qid=28&SID=D5hmJRulTs4crjeQmvg&search_mode=CombineSearches&update_back2search_link_param=yes) #5 AND #4 AND #3 AND #2 AND #1  *Índices=SCI-EXPANDED, SSCI, A&HCI, CPCI-S, CPCI-SSH, BKCI-S, BKCI-SSH, ESCI, CCR-EXPANDED, IC Período de tiempo=Todos los años*   \| # 6 \| [**327**](https://apps.webofknowledge.com/summary.do?product=WOS&doc=1&qid=28&SID=D5hmJRulTs4crjeQmvg&search_mode=CombineSearches&update_back2search_link_param=yes) \| #5 AND #4 AND #3 AND #2 AND #1  *Índices=SCI-EXPANDED, SSCI, A&HCI, CPCI-S, CPCI-SSH, BKCI-S, BKCI-SSH, ESCI, CCR-EXPANDED, IC Período de tiempo=Todos los años* \| [Editar](https://apps.webofknowledge.com/WOS_AdvancedSearch_input.do?product=WOS&SID=D5hmJRulTs4crjeQmvg&search_mode=AdvancedSearch&replaceSetId=6&editState=init) \|  \|  \| \| --- \| --- \| --- \| --- \| --- \| --- \| \|  \|  \|  \|  \|  \|  \| \| # 5 \| [**1.387.467**](https://apps.webofknowledge.com/summary.do?product=WOS&doc=1&qid=17&SID=D5hmJRulTs4crjeQmvg&search_mode=AdvancedSearch&update_back2search_link_param=yes) \| TS=(“biomarker*” OR “biological factor*” OR “nerve tissue protein” OR “nerve regeneration” OR “pain measurement” OR “glial cell line-derived neurotrophic factor” OR “glial cell line-derived neurotrophic factor receptor*” OR “brain-derived neurotrophic factor” OR “nerve growth factor receptor*” OR “receptor trkB” OR “nerve growth factor*” OR “neurotrophin*” OR “receptor trkA” OR “receptor trkC” OR “neuropeptide*” OR “cytokine*” OR “interleukin*” OR “interleukin receptor” OR “macrophage*” OR “immunology*” OR “glial cell*” OR “astrocyte*” OR “schwann cell*” OR “microglia” OR “oligodendroglia*” OR “satellite cell*”)  *Índices=SCI-EXPANDED, SSCI, A&HCI, CPCI-S, CPCI-SSH, BKCI-S, BKCI-SSH, ESCI, CCR-EXPANDED, IC Período de tiempo=Todos los años* \| [Editar](https://apps.webofknowledge.com/WOS_AdvancedSearch_input.do?product=WOS&SID=D5hmJRulTs4crjeQmvg&search_mode=AdvancedSearch&replaceSetId=5&editState=init) \|  \|  \| \|  \|  \|  \|  \|  \|  \| \| # 4 \| [**4.273.448**](https://apps.webofknowledge.com/summary.do?product=WOS&doc=1&qid=25&SID=D5hmJRulTs4crjeQmvg&search_mode=AdvancedSearch&update_back2search_link_param=yes) \| TS= (“Physical therapy modalities” OR “physical therap*” OR “physiotherap*” OR "Musculoskeletal manipulations" OR "Manual therap*” OR “exercise therapy” OR “exercise*” OR “run*” OR “swim*” OR “strength*” OR “endurance” OR “resistance” OR “physical conditioning” OR "neural tension technique*" OR “neural mobilization*” OR “neural mobilisation” OR “neural stretching” OR “neural gliding” OR “massage*” OR “massotherapy” OR “peripheral nerve therapy” OR "Electric Stimulation Therapy" OR electrotherap* OR “TENS” OR “Acupuncture” OR “dry needling” OR "manipulation*” OR ”mobilization*" OR "mobilisation*")  *Índices=SCI-EXPANDED, SSCI, A&HCI, CPCI-S, CPCI-SSH, BKCI-S, BKCI-SSH, ESCI, CCR-EXPANDED, IC Período de tiempo=Todos los años* \| [Editar](https://apps.webofknowledge.com/WOS_AdvancedSearch_input.do?product=WOS&SID=D5hmJRulTs4crjeQmvg&search_mode=AdvancedSearch&replaceSetId=4&editState=init) \|  \|  \| \|  \|  \|  \|  \|  \|  \| \| # 3 \| [**642.424**](https://apps.webofknowledge.com/summary.do?product=WOS&doc=1&qid=23&SID=D5hmJRulTs4crjeQmvg&search_mode=AdvancedSearch&update_back2search_link_param=yes) \| TS=(“neuropathic pain” OR"neuralgia" OR “neuropathic*” OR “sciatica” OR “pain”)  *Índices=SCI-EXPANDED, SSCI, A&HCI, CPCI-S, CPCI-SSH, BKCI-S, BKCI-SSH, ESCI, CCR-EXPANDED, IC Período de tiempo=Todos los años* \| [Editar](https://apps.webofknowledge.com/WOS_AdvancedSearch_input.do?product=WOS&SID=D5hmJRulTs4crjeQmvg&search_mode=AdvancedSearch&replaceSetId=3&editState=init) \|  \|  \| \|  \|  \|  \|  \|  \|  \| \| # 2 \| [**1.380.208**](https://apps.webofknowledge.com/summary.do?product=WOS&doc=1&qid=3&SID=D5hmJRulTs4crjeQmvg&search_mode=AdvancedSearch&update_back2search_link_param=yes) \| TS=("nerve block method*" OR "nerve crush" OR "nerve constriction" OR "nerve crush" OR "nerve cut" OR "nerve constriction" OR "nerve injury" OR "nerve ligation" OR "constriction pathologic*" OR "chronic constriction injury" OR "peripheral neuropathy" OR "nerve inflammation" OR "neuropathy" OR "diabetic*" OR "chemotherapy" OR "metabolic*")  *Índices=SCI-EXPANDED, SSCI, A&HCI, CPCI-S, CPCI-SSH, BKCI-S, BKCI-SSH, ESCI, CCR-EXPANDED, IC Período de tiempo=Todos los años* \| [Editar](https://apps.webofknowledge.com/WOS_AdvancedSearch_input.do?product=WOS&SID=D5hmJRulTs4crjeQmvg&search_mode=AdvancedSearch&replaceSetId=2&editState=init) \|  \|  \| \|  \|  \|  \|  \|  \|  \| \| # 1 \| [**11.064.724**](https://apps.webofknowledge.com/summary.do?product=WOS&doc=1&qid=2&SID=D5hmJRulTs4crjeQmvg&search_mode=AdvancedSearch&update_back2search_link_param=yes) \| TS= ("animal*" OR "mice" OR "rat*" OR "transgenic mice")  *Índices=SCI-EXPANDED, SSCI, A&HCI, CPCI-S, CPCI-SSH, BKCI-S, BKCI-SSH, ESCI, CCR-EXPANDED, IC Período de tiempo=Todos los años* \|  \|  \|  \|   **EMBASE:**  **No.**  **Query**  **Results**  **2,901,286**  **#90**  **#30** OR **#31** OR **#32** OR **#33** OR **#34** OR **#35** OR **#36** OR **#37** OR **#38** OR **#39** OR **#40** OR **#41** OR **#42** OR **#43** OR **#44** OR **#45** OR **#46** OR **#47**OR **#48** OR **#49** OR **#50** OR **#51** OR **#52** OR **#53** OR **#54** OR **#55** OR **#56** OR **#57** OR **#58**  **1,308,928**  **#89**  **#25** OR **#26** OR **#27** OR **#28** OR **#29**  **2,305,231**  **#88**  **#8** OR **#9** OR **#10** OR **#11** OR **#12** OR **#13** OR **#14** OR **#15** OR **#16** OR **#17** OR **#18** OR **#19** OR **#20** OR **#21** OR **#22** OR **#23** OR **#24**  **12,278,126**  **#87**  **#1** OR **#2** OR **#3** OR **#4** OR **#5** OR **#6** OR **#7**  **11,838**  **#86**  **'cytokine receptor'**  **702**  **#85**  **'glial cell line derived neurotrophic factor receptor'**  **4,512**  **#84**  **'nerve growth factor receptor'**  **6,305**  **#83**  **'satellite cell'**  **21,647**  **#82**  **'oligodendroglia'**  **53,164**  **#81**  **'microglia'**  **17,266**  **#80**  **'schwann cell'**  **69,005**  **#79**  **'astrocyte'**  **34,553**  **#78**  **'glia cell'**  **1,262,931**  **#77**  **'immunology'**  **370,799**  **#76**  **'macrophage'**  **1,515**  **#75**  **'cytokine receptor antagonist'**  **3,402**  **#74**  **'interleukin receptor'**  **669,310**  **#73**  **interleukin**  **523,111**  **#72**  **'cytokine'**  **56,941**  **#71**  **'neuropeptide'**  **18,438**  **#70**  **'neurotrophin'**  **5,329**  **#69**  **'brain derived neurotrophic factor receptor'**  **3,217**  **#68**  **'protein tyrosine kinase a'**  **1,033**  **#67**  **'receptor trkb'**  **33,151**  **#66**  **'nerve growth factor'**  **39,314**  **#65**  **'brain derived neurotrophic factor'**  **9,029**  **#64**  **'glial cell line derived neurotrophic factor'**  **8,865**  **#63**  **'pain measurement'**  **26,469**  **#62**  **'nerve regeneration'**  **44**  **#61**  **'nerve tissue protein'**  **4,816**  **#60**  **'biological factor'**  **291,640**  **#59**  **'biological marker'**  **93,459**  **#58**  **'mobilization'**  **9,272**  **#57**  **mobilisation**  **144,443**  **#56**  **manipulation***  **674**  **#55**  **'dry needling'**  **49,637**  **#54**  **'acupuncture'**  **16,019**  **#53**  **tens**  **85,616**  **#52**  **'electrostimulation'**  **14,461**  **#51**  **'electrotherapy'**  **2**  **#50**  **'peripheral nerve therapy'**  **23,397**  **#49**  **'massage'**  **11**  **#48**  **'neural gliding'**  **3**  **#47**  **'neural stretching'**  **15**  **#46**  **'neural mobilisation'**  **113**  **#45**  **'neural mobilization'**  **2**  **#44**  **'neural tension technique'**  **1,130**  **#43**  **'physical conditioning'**  **1,195,994**  **#42**  **resistance**  **45,855**  **#41**  **'endurance'**  **395,263**  **#40**  **'strength'**  **54,887**  **#39**  **swim***  **47,871**  **#38**  **'treadmill'**  **337,640**  **#37**  **run***  **568,836**  **#36**  **exercise***  **33,694**  **#35**  **'kinesiotherapy'**  **11,028**  **#34**  **'manipulative medicine'**  **5,740**  **#33**  **'manual therap*'**  **318**  **#32**  **'musculoskeletal manipulation'**  **89,891**  **#31**  **'physical therap*'**  **133,444**  **#30**  **'physiotherapy'**  **1,285,188**  **#29**  **'pain'**  **6,476**  **#28**  **'sciatica'**  **52,626**  **#27**  **'neuropathic*'**  **31,397**  **#26**  **'neuralgia'**  **40,160**  **#25**  **'neuropathic pain'**  **72,223**  **#24**  **'metabolic disorder'**  **866,443**  **#23**  **metabolic***  **886,136**  **#22**  **'chemotherapy'**  **430,426**  **#21**  **'diabetic*'**  **197,530**  **#20**  **'neuropathy'**  **307**  **#19**  **'nerve inflammation'**  **57,586**  **#18**  **'peripheral neuropathy'**  **2,469**  **#17**  **'chronic constriction injury'**  **7,254**  **#16**  **'stenosis, occlusion and obstruction'**  **102**  **#15**  **'constriction pathologic*'**  **3,051**  **#14**  **'nerve ligation'**  **54,329**  **#13**  **'nerve injury'**  **248**  **#12**  **'nerve cut'**  **228**  **#11**  **'nerve constriction'**  **15,583**  **#10**  **'nerve compression'**  **3,943**  **#9**  **'nerve crush'**  **154**  **#8**  **'nerve block method*'**  **65,081**  **#7**  **'transgenic mice'**  **8,107,007**  **#6**  **rat***  **1,177,969**  **#5**  **mice**  **82,727**  **#4**  **'transgenic mouse'**  **1,963,922**  **#3**  **'mouse'**  **6,274,624**  **#2**  **animal***  **1,372,929**  **#1**  **'animal model'**/exp OR **'animal model'**  Final del formulario |
|  |
| **Cochrane Library Search Formula.** |

1. MeSH descriptor: [Models, Animal] explode all tres
2. (“animal*”):ti,ab,kw
3. (“mice”):ti,ab,kw
4. (“mice”):ti,ab,kw
5. (“transgenic mice”):ti,ab,kw
6. (“nerve block method*”):ti,ab,kw
7. (“nerve block method*”):ti,ab,kw
8. (“nerve constriction”):ti,ab,kw
9. (“nerve crush”):ti,ab,kw
10. (“nerve cut”):ti,ab,kw
11. (“nerve constriction”):ti,ab,kw
12. ("Nerve injury"):ti,ab,kw
13. (“nerve ligation”):ti,ab,kw
14. (“constriction pathologic*”):ti,ab,kw
15. (“chronic constriction injury”):ti,ab,kw
16. (“peripheral neuropathy”):ti,ab,kw
17. (“nerve inflammation”):ti,ab,kw
18. (“neuropathy”):ti,ab,kw
19. ("diabetic*"):ti,ab,kw
20. ("chemotherapy*"):ti,ab,kw
21. ("metabolic*"):ti,ab,kw
22. MeSH descriptor: [Neuralgia] explode all tres
23. (“neuropathic pain”):ti,ab,kw
24. (“neuropathic*”):ti,ab,kw
25. (“sciatica”):ti,ab,kw
26. (“pain”):ti,ab,kw
27. MeSH descriptor: [Physical Therapy Modalities] explode all tres
28. (“physical therapy”):ti,ab,kw
29. (“physiotherapy”):ti,ab,kw
30. ("Musculoskeletal manipulations"):ti,ab,kw
31. (Manual therap*):ti,ab,kw
32. (“exercise therapy”):ti,ab,kw
33. (“exercise*”):ti,ab,kw
34. (“run*”):ti,ab,kw
35. (“swim*”):ti,ab,kw
36. (“strength*”):ti,ab,kw
37. (“endurance”):ti,ab,kw
38. (“resistance”):ti,ab,kw
39. (“physical conditioning”):ti,ab,kw
40. ("neural tension technique"):ti,ab,kw
41. (“neural mobilization*”):ti,ab,kw
42. (“neural stretching”):ti,ab,kw
43. (“neural stretching”):ti,ab,kw
44. (“massage*”):ti,ab,kw
45. (“massotherapy”):ti,ab,kw
46. (“peripheral nerve therapy”):ti,ab,kw
47. MeSH descriptor: [Electric Stimulation Therapy] explode all tres
48. (electrotherap*):ti,ab,kw
49. (“TENS”):ti,ab,kw
50. MeSH descriptor: [Acupuncture] explode all tres
51. (“dry needling”):ti,ab,kw
52. (manipulation):ti,ab,kw
53. (mobilization*):ti,ab,kw
54. ("mobilisation*"):ti,ab,kw
55. (“biomarker*”):ti,ab,kw
56. (“biological factor*”):ti,ab,kw
57. (“nerve tissue protein”):ti,ab,kw
58. (“nerve regeneration”):ti,ab,kw
59. (“pain measurement”):ti,ab,kw
60. (“glial cell line-derived neurotrophic factor”):ti,ab,kw
61. (“glial cell line-derived neurotrophic factor receptor*”):ti,ab,kw
62. (“receptor trkB”):ti,ab,kw
63. (“nerve growth factor*”):ti,ab,kw
64. (“neurotrophin*”):ti,ab,kw
65. (“receptor trkA”):ti,ab,kw
66. (“receptor trkC”):ti,ab,kw
67. (“neuropeptide*”):ti,ab,kw
68. (“cytokine*”):ti,ab,kw
69. (“interleukin*”):ti,ab,kw
70. (“interleukin receptor”):ti,ab,kw
71. (“macrophage*”):ti,ab,kw
72. (“immunology*”):ti,ab,kw
73. (“glial cell*”):ti,ab,kw
74. (“astrocyte*”):ti,ab,kw
75. (“schwann cell*”):ti,ab,kw
76. (“microglia”):ti,ab,kw
77. (“oligodendroglia*”):ti,ab,kw
78. (“satellite cell*”):ti,ab,kw

{OR #1-#5} AND {OR #6-#26} AND {OR #27-#54} AND {OR #55-#78}
